# Standardized quantum transistor block enables differentiable learning on gait dynamics

**DOI:** 10.1038/s41598-026-40424-7

**Published:** 2026-02-18

**Authors:** Javier Villalba-Díez, Joaquín Ordieres-Meré

**Affiliations:** 1 Fakultät Wirtschaft, Hochschule Heilbronn, Max-Planck-Str.39, 74081 Heilbronn, Baden-Württemberg Germany; 2 Department of Mechanical Engineering, Universidad de La Rioja, Edificio Departamental, c/ San José de Calasanz, 31, 26004 Logroño, La Rioja Spain; 3 Escuela Técnica Superior de Ingenieros Industriales, Universidad Politécnica de Madrid, C/ José Gutierrez Abascal, 2, 28006 Madrid, Spain

**Keywords:** Quantum transistor, Variational quantum circuits, Quantum signal processing, Gait analysis, Hybrid AI–quantum pipelines, Standardization, Engineering, Mathematics and computing, Physics

## Abstract

We introduce the *Quantum Transistor* (QT), a standardized variational quantum building block inspired by the operating-point and gain semantics of classical transistors. The QT is specified as a two-qubit template (gate *g*, channel *t*), but the experiments reported here instantiate the *non-entangling special case* in which *g* is deterministically prepared in $|1\rangle$ and is not reused; consequently, the controlled-$R_y(\phi )$ bias reduces to an unconditional single-qubit $R_y(\phi )$ on the channel qubit *t*. In this instantiation the QT implements an analytically tractable, bounded scalar nonlinearity $s\mapsto \langle Z_t\rangle$ with closed-form gain and saturation, and the full QT stack is efficiently classically simulable (a shallow classical contraction composed with bounded trigonometric scalar nonlinearities). We evaluate this standardized, single-qubit-effective QT stack on subject-aware gait classification with HyperBand and grouped cross-validation. In a locked-out 3-fold grouped protocol, the QT network reaches the mean test accuracy 0.960 and the mean F1 0.931. Strong classical baselines (CNN and Transformer encoders operating directly on the same spectrogram windows from which the QT’s 8-D inputs are derived) achieve F1 in the 0.962–0.964 range. Although the present QT stack (in its non-entangling, efficiently classically simulable instantiation) is outperformed by parameter-matched classical baselines, the objective of this paper is to validate a standardized, analyzable *quantum-layer primitive* rather than to claim superiority on this dataset. Accordingly, we position the evaluated system as a quantum-inspired, hybrid structured model study and we do not claim quantum advantage in this manuscript. The QT’s fixed port contract, closed-form gain/saturation, and constant-depth instruction template enable portable compilation, lightweight conformance tests (midpoint/slope/noise contraction), and predictable shot/latency budgeting. These properties are directly relevant in settings where a quantum co-processor is available as part of the system (e.g., integration with quantum sensing or other quantum-data pipelines). We therefore contextualize accuracy with strong classical baselines while outlining the block-level extensions (trainable biasing, pooling, richer encodings, and genuinely data-dependent gating) needed to close the remaining performance gap.

## Introduction

Transistors transformed computing by providing a *standardized*, composable primitive with a clear input–output semantics, a controllable notion of gain, and robust fabrication pathways^1^. At the level of the computational model, quantum computing already enjoys an analogous form of standardization: the circuit model, Pauli operators, and a small set of universal gate families are widely agreed upon and underpin most hardware and software stacks. The present work concerns a different layer, namely the *block-level* primitives that are used as building bricks inside variational quantum algorithms. At this level, most quantum machine learning models are still assembled from bespoke variational circuits whose roles differ between tasks and whose interfaces are rarely specified beyond code-level detail^2,3^. This relative lack of block-level standardization hampers re-use, formal analysis, and hardware co-design. In this work, we take a step toward a quantum analogue of the transistor: a small, self-contained, differentiable *Quantum Transistor* (QT) block with explicit *gating* semantics, a well-defined *gain* profile, and an electrical metaphor that enables system-level design rather than circuit-by-circuit craftsmanship.

Classical transistors (BJT or MOSFET) are three-terminal devices whose gate/base terminal *biases* a channel and thus modulates the current between the remaining two terminals^4^. Designers set a *quiescent operating point* (Q-point) via a DC bias so that small AC variations at the input produce amplified variations at the output. The local small-signal *transconductance* is $g_m \equiv \partial I_{\textrm{out}}/\partial V_{\textrm{in}}$ (units: S), evaluated at the Q-point. The input–output transfer curve is *saturating*: Outside a mid-slope region (linear regime), the device attaches near the supply rails. This vocabulary, operating point and small signal gain, enables system-level reasoning (gain staging, noise budgeting, stability), which we mirror for the QT.

In brief, a QT is specified as a two-qubit template with a *gate* qubit *g* and a *channel* qubit *t*: *s*-scaled single-qubit rotations act on *t*, a bias interaction is expressed as a controlled $R_y(\phi )$ from *g* to *t*, and the block outputs $y(s)=\langle Z_t\rangle \in [-1,1]$. Importantly, the experiments in this paper use the special case $g=|1\rangle$ (and do not reuse *g*), so the bias interaction is operationally identical to an unconditional $R_y(\phi )$ on *t* and no entanglement is generated; consequently, the implemented QT network is mathematically equivalent to a classical stack of bounded analytic scalar nonlinearities composed with the shallow contraction layer. We retain the explicit gate wire in the *definition* of the QT to provide a standardized interface for future data-dependent gating variants (e.g., shared gate qubits or learned gate encodings) where the same template would generate genuine two-qubit entanglement without changing the software contract. The exact unitary, closed-form transfer $s\mapsto y(s)$, and small-signal transconductance appear in Sect. "Quantum transistor" (Eqs. (19)–(14)).

Standardization at the block level is not merely aesthetic. It brings three concrete advantages. *(i) Interface clarity.* Declaring ports (*one real in, one expectation out*), parameter vectors, and initialization/measurement conventions makes blocks *modular*: the same QT can be dropped into different stacks, data modalities, or hardware backends without re-deriving the basics. *(ii) Physical analyzability.* Because the QT is only two qubits deep and uses a minimal gate set, it admits compact expressions for its *transconductance* $g_q(s)=\partial \langle Z\rangle /\partial s$ and its *saturation behavior*. These quantities provide exactly the kind of mid-level, device-agnostic reasoning that classical electronics relies on (gain curves, operating regions). *(iii) Hardware viability.* With few entangling gates and strictly local rotations, QTs map cleanly to noisy, near-term processors and to simulators; they also lend themselves to vendor-agnostic libraries of primitives.

We evaluated QTs on a real clinically meaningful problem: *gait state recognition for multiple sclerosis patients*. Gait segments exhibit a rich time–frequency structure and, more importantly, require *subject-aware* validation to prevent identity leakage. The task is representative of a wider class of biosignal problems: low latency, safety-critical inference on short windows, where compact models and system-level reliability matter as much as raw precision^5^. Our pipeline mirrors best practice in statistical learning: strict grouped cross-validation by subject/session, calibrated thresholds chosen on validation folds to maximize F1 (rather than hard 0.5 cutoffs), and a held-out reporting protocol^6^. In addition to the quantum model, we train strong baselines on exactly the same spectrogram windows and subject-grouped splits to bound performance and contextualize the quantum results. The baselines consume the full $40\times 12$ spectrogram-like tensors, whereas the QT stack only sees the eight-dimensional contracted features $h\in [-1,1]^8$ produced by Eq. (22) from those same tensors.

**Table 1:** Classical–quantum analogy used in this work.

Classical transistor (amplifier view)	Quantum transistor (this work)
Input (gate/base voltage) $V_{\textrm{in}}$	Normalized scalar feature $s\in [-1,1]$
DC bias / Q-point	Bias angle $\phi$ applied as $\textrm{CRY}^{(g\rightarrow t)}(\phi )$
Output (current/voltage)	Readout $y(s)=\langle Z_t\rangle \in [-1,1]$
Small-signal transconductance $g_m=\partial I_{\textrm{out}}/\partial V_{\textrm{in}}$	$g_q(s)=\partial y/\partial s$ (Eq. (14))
Saturating transfer curve	Bounded Bloch-sphere transfer $s\mapsto y(s)$ (Eq. (11))

This paper is motivated by four research questions. *RQ1:* Can a standardized, two-qubit QT block implement a useful, transistor-like *gating and amplification* nonlinearity with stable gradients suitable for end-to-end learning? *RQ2:* How should QTs be *stacked*-in depth and fan-in-to form expressive yet shallow networks that remain trainable under realistic resource constraints? *RQ3:* On a subject-aware gait classification task, does a QT network achieve competitive generalization compared with classical baselines when assessed under identical cross-validation and calibration protocols? *RQ4:* Which block-level design choices (e.g., fixed vs. learnable bias angle, number of pre-bias rotations, pooling of multi-block outputs) most strongly affect the trade-off between expressivity, stability, and hardware cost?

To address these questions we instantiate a three-stage QT network. A small linear contraction maps each high-dimensional segment into eight normalized signals in $[-1,1]$; Layer 1 comprises four QTs (8 qubits) and returns four scalar expectations; Layer 2 comprises three QTs (6 qubits) and returns three scalars; Layer 3 comprises two QTs (4 qubits) and returns two scalars, from which we read a single *logit*. TThis layout intentionally keeps entangling depth modest. In the present single-head instantiation, the logit depends only on a single propagated chain through the stack (Sect. "Robustness to noise and calibration"), so the reported experiments do not yet probe multi-path learning across the full 4–3–2 scaffold. The parameters of each QT include a vector of rotation scalings (controlling sensitivity to *s*) and a bias-like controlled-rotation angle; in our prototype the bias is fixed, highlighting both the strengths and the limitations of a non-learned operating point. Training proceeds with a class-weighted logistic loss to handle label imbalance; gradients are exact via parameter-shift; and we employ Adam with learning rate selected by HyperBand. Importantly, the decision threshold is not fixed; it is *calibrated* on the validation set to optimize F1, and the resulting threshold is then used-unchanged-on the test fold.

Hyperparameter search explores the number of per-block rotation parameters and the learning rate; the best configuration in our runs uses five rotation parameters per block and a learning rate of approximately $4.3\times 10^{-4}$. We then *freeze* this configuration and perform a fresh grouped 3-fold evaluation. The quantum model attains mean test accuracy 0.960 and mean F1 0.931, with an average confusion matrix indicating low false positives and modest false negatives under the calibrated thresholds. The classical baselines, trained under the same protocol, achieve F1 in the 0.962–0.964 range. While the QT stack does not yet surpass the best classical model on this dataset, we use gait classification primarily as a realistic *integration test* for a standardized QT primitive: it stresses the aspects that matter for deployment (bounded I/O ranges, validation-threshold calibration, predictable resource budgets, and circuit-template portability) rather than optimizing solely for maximal F1. Concretely, the QT layer has a backend-portable, constant-depth template and an analyzable gain profile, which supports hardware/software co-design (compilation, scheduling, calibration, and conformance testing) and makes the block usable as a plug-in component when a quantum co-processor is present (e.g., co-located with quantum sensors or other quantum data sources). Accordingly, we report strong classical baselines to contextualize current accuracy and explicitly avoid any claim of quantum superiority; the roadmap we outline—trainable biasing to place operating points, pooling of multiple last-layer heads rather than a single-logit readout, richer encodings (including data re-uploading), and genuinely data-dependent gating variants—describes what is required to close the present performance gap while preserving the same block-level interface.

Beyond accuracy, the QT perspective yields qualitative benefits that practitioners will recognize. By measuring per-epoch $\langle Z\rangle$ trajectories in each block, we can *see* the model bias itself away from saturation and stabilize in a mid-slope region-precisely the transistor-like behavior the metaphor suggests. This introspectability is not a luxury: it supports debugging, calibration, and trust in safety-critical pipelines. Moreover, the block abstraction enables clean separation of concerns. Application teams can design pre-processing and choose operating points; hardware teams can refine decompositions, native gate choices, and noise mitigation for the fixed QT schema; learning teams can study optimization, calibration, and regularization effects at the block and network levels.

From a standardization vantage point, we advocate cataloguing QTs with a minimal schema: *ports* (in: $s\in [-1,1]$; out: $y=\langle Z\rangle$), *parameters* (rotation scalings; optional bias), *gateset* (*X*, $R_x$, $R_y$, $R_z$, and a single controlled $R_y$), *init* ($|1\rangle \!\otimes \!|0\rangle$), *forward* (unitary followed by *Z* measurement), and *backward* (parameter-shift). This is specific enough for compilation and verification, yet generic enough to be vendor-agnostic. A small library of such primitives would move our community toward interoperable, testable quantum *systems* and away from one-off circuits.

Finally, a brief human note. The transistor metaphor is not window dressing; it is a practical bridge between disciplines. It equips algorithm designers, hardware engineers, and application scientists with a shared language, gain, operating point, saturation, that reduces friction of collaboration. The present study offers evidence that this language can be made precise in quantum learning, that it produces competitive performance on a meaningful task, and that it opens a roadmap where improvements are expressed *at the block level*. In the pages that follow, we formalize the QT mathematically, describe the stacked architecture and training protocol, present results and ablations, and distill design lessons for future hardware-aware quantum learning.

Our contributions can be summarized as follows:

1. Building on established “quantum neuron” and feature-map VQC constructions (parameterized single-qubit rotations followed by measurement), we package a concrete *Quantum Transistor* (QT) block with an explicit port contract ($s\in [-1,1]\mapsto y=\langle Z\rangle \in [-1,1]$), a fixed gate ordering (bias-last), and an implementation-compatible template. We derive the closed-form transfer and small-signal transconductance, enabling analytic verification and fast simulation.
2. We introduce an electronics-style characterization of this primitive (operating point, saturation, and small-signal gain) and use it to motivate a layer-wise *gain-budgeting* view for stacking QTs while preserving stable gradients and bounded outputs.
3. We specify a minimal engineering-style *block specification* for QT implementations (ports, initialization/measurement conventions, parameter-shift compatibility, and compilation assumptions for the bias interaction), together with simple *conformance tests* (midpoint, slope, and monotone noise contraction) that support portability across software stacks and hardware backends.
4. We evaluate the resulting QT stack end-to-end on a subject-grouped gait task with threshold calibration, provide budget-matched classical baselines, and release a reproducible pipeline; we explicitly delimit claims and do not assert conceptual novelty of the underlying “few rotations + measurement” scalar activation mechanism.

The remainder of the paper is organized as follows. Section "Background and related work" situates our work within quantum learning and hardware-efficient design. Section "Quantum transistor" formalizes the QT mathematically, details the stacked architecture and training regime, and specifies the evaluation protocol. Section  "Classical baselines and data collection process", presents the classical baselines and the data collection process. Section "Results and analysis" presents empirical results, ablations, and per-layer analyses. Section "Discussion" discusses implications, limitations, and design lessons. Section "Conclusion and future work" concludes with a roadmap for block-level standardization in quantum machine learning.

## Background and related work

Variational quantum learning (VQL) places tunable parameters inside a parameterized unitary and optimizes them against a classical objective computed from measurement statistics^2^. Formally, let $\rho _{\textrm{in}}$ be a prepared *n*-qubit input state (possibly depending on classical data *x* via an *encoding* *E*(*x*)), and let

1 $$\begin{aligned} U(\boldsymbol{\theta }) \;=\; \prod _{\ell =1}^{L} \exp \!\big (-i\,\theta _\ell H_\ell \big ) \end{aligned}$$

be a depth-*L* unitary with generators $\{H_\ell \}$ drawn from a fixed, hardware-efficient gate set. An observable *M* (or a small set $\{M_j\}$) defines the model output through expectations.

2 $$\begin{aligned} f_{\boldsymbol{\theta }}(x)\;=\; \textrm{Tr}\!\left[ M\,U(\boldsymbol{\theta })\,E(x)\,\rho _{\textrm{in}}\,E(x)^\dagger \,U(\boldsymbol{\theta })^\dagger \right] . \end{aligned}$$

Training proceeds by minimizing a classical loss $\mathcal {L}\big (f_{\boldsymbol{\theta }}(x),y\big )$ over data (*x*, *y*) using gradient-based optimizers^7^. For rotation-generated gates ($H_\ell$ with two eigenvalues $\pm \tfrac{1}{2}$), the *parameter-shift rule*^8^ provides exact derivatives without back-propagating through stochastic measurement:

3 $$\begin{aligned} \frac{\partial }{\partial \theta _\ell } f_{\boldsymbol{\theta }}(x) \;=\;\frac{1}{2}\Big [f_{\boldsymbol{\theta }^{(\ell ,+)}}(x)-f_{\boldsymbol{\theta }^{(\ell ,-)}}(x)\Big ],\qquad \boldsymbol{\theta }^{(\ell ,\pm )} = \boldsymbol{\theta }\pm \tfrac{\pi }{2}\,\textbf{e}_\ell . \end{aligned}$$

Here $\textbf{e}_\ell$ denotes the standard basis vector in parameter space, i.e., the vector with a 1 in position $\ell$ and zeros elsewhere.

Despite this clean calculus and the underlying standardization provided by the circuit model and commonly used gate sets, two gaps remain at the level of reusable variational blocks and system-level design:

- *Interface ambiguity*^9^. A variational quantum algorithm “block” should be specified as a *typed* family of completely positive trace-preserving maps with an explicit measurement following Eq. 2^10^. Reproducibility requires that the *contract* expose: (a) the domain/codomain of the ports (classical input scaling; output range), (b) the encoding family $E(\cdot )$, (c) the generator spectra of $\{H_\ell \}$ (so gradient rules such as parameter-shift apply), (d) the measurement operators and estimators, and (e) the native gateset/compilation assumptions. Despite these, two implementations with the same symbol $f_{\boldsymbol{\theta }}(x)$ can differ in gradients, noise profiles, and even output ranges, defeating modular composition, testing, and hardware co-design.
- *System design without primitives.* In analog design a primitive is characterized by a transfer *y*(*u*), an operating point $u_0$, a *small-signal* gain $g=\partial y/\partial u|_{u_0}$, bounded output and noise figures. Typical variational quantum circuits do not declare an analogous block-level transfer $h(\cdot ;\boldsymbol{\theta })$ with an operating region and gain. For a depth-*D* stack with layer maps $\textbf{y}^{(\ell )}=\textbf{h}^{(\ell )}(\textbf{y}^{(\ell -1)})$, the chain-rule bound
  4 $$\begin{aligned} \Vert \nabla _x \textbf{y}^{(D)}\Vert \;\le \;\Big (\prod _{\ell =1}^D \Vert J^{(\ell )}\Vert \Big )\,\Vert \nabla _x \textbf{y}^{(0)}\Vert \end{aligned}$$
  is therefore uncontrolled because $\Vert J^{(\ell )}\Vert$ depends on undocumented encoder scales, rotation spectra, and readouts. The result is either vanishing/exploding gradients or opaque robustness under noise^11–14^. A standardized primitive, e.g., a bounded map $s\mapsto y(s)\in [-1,1]$ with an explicit small-signal slope $g_q(s_0)=\partial y/\partial s\,|_{s_0}$ and a simple noise model $y_{\text {noisy}}=\lambda \,y+t$, provides the mid-level quantities (gain, operating region, saturation) needed for predictable layer-wise design and hardware/resource budgeting.

We adopt the standard amplifier vocabulary (Q-point, small-signal transconductance) introduced in Sect. "Introduction" and summarized in Table 1, using it to reason about gain staging and operating regions in the QT stack.

The QT we advocate pursues a middle ground: a two-qubit, differentiable primitive with an explicit input–output contract, analytic gain, and hardware-efficient depth. Before formalizing the QT, we summarize geometric and algebraic intuitions that motivate its design.

Any pure single-qubit state can be represented by a Bloch vector $\textbf{v}\in \mathbb {R}^3$^15^ with $\Vert \textbf{v}\Vert =1$, and any unitary $U\in SU(2)$ acts as a real rotation $\mathcal {R}(U)\in SO(3)$: $\textbf{v}\mapsto \mathcal {R}(U)\textbf{v}$. For the Pauli-*Z* expectation one simply reads the *z*-component,

5 $$\begin{aligned} \langle Z\rangle \;=\; v_z. \end{aligned}$$

Elementary rotations about coordinate axes correspond to

6 $$\begin{aligned} \mathcal {R}_x(\theta )&= \begin{bmatrix} 1 & 0 & 0\\ 0 & \cos \theta & -\sin \theta \\ 0 & \sin \theta & \cos \theta \end{bmatrix},\quad \mathcal {R}_y(\theta ) = \begin{bmatrix} \cos \theta & 0 & \sin \theta \\ 0 & 1 & 0\\ -\sin \theta & 0 & \cos \theta \end{bmatrix}, \mathcal {R}_z(\theta ) = \begin{bmatrix} \cos \theta & -\sin \theta & 0\\ \sin \theta & \cos \theta & 0\\ 0 & 0 & 1 \end{bmatrix}. \end{aligned}$$

If the rotation angles are made *proportional* to a real, normalized input $s\in [-1,1]$, say $\alpha s$, $\beta s$, $\gamma s$, then the *Z*-expectation after a short sequence of $R_y$, $R_x$, $R_z$ rotations becomes a trigonometric polynomial in *s*. Appending a *controlled* rotation from a gate qubit set to $|1\rangle$ performs a bias-like shift of the operating point, exactly analogous to transistor biasing.

A single QT uses two qubits: a *control* (gate) *g* and a *channel* (target) *t*. The contract is:

**Table:** 

*Input port:*	one real$s\in [-1,1]$(normalized feature)
*Parameters:*	$\boldsymbol{\theta }=(\theta _1,\theta _2,\theta _3,\dots )$(rotation scalings)
*Bias:*	fixed angle$\phi$applied as a controlled$R_y(\phi )$from$g\rightarrow t$
*Output port:*	scalar$y(s)=\langle Z_t\rangle \in [-1,1]$

For concreteness, consider the minimal three-parameter instance

7 $$\begin{aligned} U_{\textrm{QT}}(s;\boldsymbol{\theta },\phi )= \textrm{CRY}(\phi )^{(g\rightarrow t)}\,R_z^{(t)}(\gamma s)\,R_x^{(t)}(\beta s)\,R_y^{(t)}(\alpha s),\quad \text {with state } |10\rangle \text { as input.} \end{aligned}$$

We initialize *g* to $|0\rangle$ and *t* to $|0\rangle$, so $X^{(g)}$ prepares $g=|1\rangle$ and activates the control on the subsequent controlled-$R_y(\phi )$. Writing the channel Bloch vector before the controlled rotation as $\textbf{v}'(s)$ and applying Eq. (6) in sequence to $\textbf{v}_0=(0,0,1)$ yields

8 $$\begin{aligned} \textbf{v}_1(s)&= \mathcal {R}_y(\alpha s)\textbf{v}_0 = \big (\sin (\alpha s),\,0,\,\cos (\alpha s)\big ), \end{aligned}$$

9 $$\begin{aligned} \textbf{v}_2(s)&= \mathcal {R}_x(\beta s)\textbf{v}_1(s) = \big (\sin (\alpha s),\, -\cos (\alpha s)\sin (\beta s),\, \cos (\alpha s)\cos (\beta s)\big ), \end{aligned}$$

10 $$\begin{aligned} \textbf{v}_3(s)&= \mathcal {R}_z(\gamma s)\textbf{v}_2(s) \nonumber \\&= \big (\sin (\alpha s)\cos (\gamma s) + \cos (\alpha s)\sin (\beta s)\sin (\gamma s),\; \sin (\alpha s)\sin (\gamma s) \nonumber \\&\quad- \cos (\alpha s)\sin (\beta s)\cos (\gamma s),\; \cos (\alpha s)\cos (\beta s)\big ). \end{aligned}$$

Since the bias gate is the controlled rotation

$$\textrm{CRY}^{(g\rightarrow t)}(\phi ) = |0\rangle \!\langle 0|_g \otimes I_t \;+\; |1\rangle \!\langle 1|_g \otimes R_y^{(t)}(\phi ),$$

and the control qubit is deterministically prepared as $|1\rangle _g$ before the bias is applied, its action on the channel reduces to an *unconditional* $R_y(\phi )$ on *t*. In the present prototype, therefore, the implemented map on the channel qubit is *exactly* the same as that of a single-qubit “bias-last” block, and no entanglement with *g* is generated. We nevertheless keep an explicit gate wire in the definition of a Quantum Transistor for three reasons: (i) it mirrors the three-terminal structure of a classical transistor and makes it straightforward to generalize to data-dependent gating where the state of *g* is nontrivial and may be *shared* across several channels; (ii) it matches hardware that already exposes native controlled rotations or calibrated two-qubit pulses, so that future variants that actually entangle *g* and *t* can reuse exactly the same block specification; and (iii) it allows us to state an entangling budget that is an *upper bound* valid also for such data-dependent extensions. For the specific experiments reported here, a compiler is free to collapse the bias into a single-qubit rotation $R_y(\phi )$ on *t* and to discard the idle control wire without changing the transfer function or the training dynamics. Therefore the post-bias Bloch vector is $\textbf{v}(s)=\mathcal {R}_y(\phi )\,\textbf{v}_3(s)$, whose *z*-component gives

11 $$\begin{aligned} \begin{aligned} y(s) &= \langle Z_t\rangle = v_z(s)= \cos \phi \,\cos (\alpha s)\cos (\beta s) \\&\quad -\, \sin \phi \Bigl [\sin (\alpha s)\cos (\gamma s) + \cos (\alpha s)\sin (\beta s)\sin (\gamma s)\Bigr ]. \end{aligned} \end{aligned}$$

The first term is an *even* function of *s* that saturates to $\pm 1$ as either $\alpha s$ or $\beta s$ approaches $\pm \tfrac{\pi }{2}$; it is scaled by $\cos \phi$ and thus suppressed when the bias approaches $\tfrac{\pi }{2}$. The bracketed term is *odd* in *s* to first order and is scaled by $\sin \phi$; it provides the main linear response around the operating point. In this sense, $\phi$ *opens* or *closes* the channel’s transconductance window, directly paralleling gate bias in a classical transistor.

*Small-slope (linear-region) gain.* Expanding Eq. (11) for small-*s* gives

12 $$\begin{aligned} y(s)\;=\;\cos \phi \;-\; (\alpha \,\sin \phi )\,s \;-\; \Big [\tfrac{1}{2}\,\cos \phi \,(\alpha ^2+\beta ^2)\;+\;\beta \gamma \,\sin \phi \Big ]\,s^2 \;+\; \mathcal {O}(s^3), \end{aligned}$$

where we used $\sin (\alpha s)=\alpha s+\mathcal {O}(s^3)$, $\cos (\alpha s)=1-\tfrac{1}{2}\alpha ^2 s^2+\mathcal {O}(s^4)$ and similarly for $\beta ,\gamma$; the first $\gamma$-dependent contribution appears at quadratic order via the cross term $\beta \gamma \,\sin \phi \,s^2$. The *transconductance* (slope) at $s=0$ is therefore

13 $$\begin{aligned} g_q(0)\;=\;\left. \frac{\partial y}{\partial s}\right| _{s=0} \;=\; -\,\alpha \,\sin \phi . \end{aligned}$$

This identity captures the essential QT semantics: the linear-region gain is jointly set by the input-scaling parameter $\alpha$ and the bias lever $\phi$. When $\phi =0$ the block is *pinched off* ($g_q(0)=0$); when $\phi =\tfrac{\pi }{2}$ it reaches maximum slope $|g_q(0)|=|\alpha |$.

*General derivative and saturation.* Differentiating Eq. (11) gives the exact transconductance for any *s*:

14 $$\begin{aligned} \begin{aligned} \frac{\partial y}{\partial s}&= -\cos \phi \Big [\alpha \sin (\alpha s)\cos (\beta s)+\beta \cos (\alpha s)\sin (\beta s)\Big ]\\&\quad -\sin \phi \Big [\alpha \cos (\alpha s)\cos (\gamma s)-\gamma \sin (\alpha s)\sin (\gamma s)\\&\quad -\alpha \sin (\alpha s)\sin (\beta s)\sin (\gamma s) +\beta \cos (\alpha s)\cos (\beta s)\sin (\gamma s)\Big ]. \end{aligned} \end{aligned}$$

Points where $\partial y/\partial s=0$ are transfer extrema (plateaus); conversely, neighborhoods where $|\partial y/\partial s|$ is large define the effective linear regime for cascading.

*Additional per-block rotations.* In practice a QT may include extra Rot gates with a shared angle $\delta s$ (i.e., a sequence $R_z(\delta s)R_y(\delta s)R_x(\delta s)$). Each such triple composes a new *SO*(3) rotation whose entries are trigonometric polynomials in *s*; hence *y*(*s*) remains a bounded trigonometric polynomial with richer harmonics. Importantly, the linear-region slope at $s=0$ still obeys Eq. (13) with $\alpha$ replaced by the *effective* *y*-axis coefficient of the composed rotation. Thus, extra re-uploading increases expressivity primarily beyond first order, while keeping the first-order gain governed by the *y*-axis scaling and the bias $\phi$.

Because Eq. (11) is a finite trigonometric polynomial in *s*, a single QT realizes a one-dimensional *Fourier feature map* with *learnable frequencies* $\{\alpha ,\beta ,\gamma ,\dots \}$ and learnable mixing controlled by $\phi$. For multiple inputs $\textbf{s}=(s_1,\dots ,s_m)$ feeding *m*/2 QTs in parallel, the layer output is a vector $\textbf{y}(\textbf{s})$ whose entries are tensor products of one-dimensional trigonometric polynomials; with *K* data re-uploads per block, the total degree in each $s_i$ is bounded by *K*.

Let $\mathcal {T}_K$ denote the set of functions representable by a QT with up to *K* re-uploads (i.e., *K* effective single-qubit rotation triplets). Then $\mathcal {T}_K$ equals the set of trigonometric polynomials of degree at most *K* in *s* after an affine reparameterization of the coefficients. Stacking layers with affine mixing of inputs (as done by the learned linear downsampler in our network) yields *mixtures of trigonometric polynomials* over linear combinations of the original features. In the small-angle regime (typical at initialization), each block behaves like a linear function plus bounded higher-order corrections, enabling gradient flow; during training, the model can *self-bias* into a mid-slope region where harmonics enrich the decision boundary.

Consider a depth-*D* cascade where layer $\ell$ implements a vector map $\textbf{y}^{(\ell )} = \textbf{h}^{(\ell )}(\textbf{y}^{(\ell -1)})$, with Jacobian $J^{(\ell )}(\textbf{y})=\partial \textbf{h}^{(\ell )} / \partial \textbf{y}$. A standard chain rule bound yields

15 $$\begin{aligned} \big \Vert \nabla _{\textbf{s}}\,y_{\text {out}} \big \Vert \;\le \; \prod _{\ell =1}^{D} \big \Vert J^{(\ell )}\big \Vert \;\cdot \;\big \Vert \nabla _{\textbf{s}}\,\textbf{y}^{(0)}\big \Vert . \end{aligned}$$

Because each QT has a bounded slope, one has the $\phi$-dependent bound

$$\big |\tfrac{\partial y}{\partial s}\big | \le |\cos \phi |\, (|\alpha |+|\beta |) + |\sin \phi |\,(2|\alpha |+|\beta |+|\gamma |) \;\le \; 2|\alpha |+2|\beta |+|\gamma |,$$

from Eq. (14), so one can select per-layer scaling to avoid both gradient vanishing (too small product) and gradient explosion (too large product). In our design, we (i) compress the classical input via a linear map to keep signals in $[-1,1]$, and (ii) restrict per-block scalings and depth to maintain a *gain budget* that supports stable training.

Two pathologies matter in practice: *flat gradients* and *high curvature*. For small random initializations with shallow, local gates, the parameter-shift gradient in Eq. (3) enjoys $\mathcal {O}(1)$ variance that does not shrink with the total number of qubits because each QT touches only two qubits and uses few entanglers. Moreover, the small-*s* expansion of Eq. (12) shows that first-order sensitivity depends on $\alpha \sin \phi$, a quantity that can be tuned away from zero at initialization by choosing $\phi \approx \pi /3$ and nonzero $\alpha$. High curvature arises when $|\alpha s|$ or $|\beta s|$ approach $\tfrac{\pi }{2}$, where $\cos (\cdot )$ crosses zero and second derivatives spike. Our schedule avoids such regimes early in training by (a) normalizing inputs, (b) *annealing* learning rates, and (c) using validation-threshold calibration (see below) so that optimization is not forced to adjust parameters only to accommodate a suboptimal fixed decision threshold.

A consolidated robustness analysis under standard noise channels (unital and non-unital) and its implications for calibration is provided in Sect. "Robustness to noise and calibration".

### From generic VQCs to standardized blocks: ports, parameters, and semantics

We advocate a minimal *specification* for hardware-agnostic QT libraries:

- *Ports.* in: $s\in [-1,1]$ (float); out: $y=\langle Z\rangle \in [-1,1]$ (float).
- *Parameters.* $\boldsymbol{\theta }\in \mathbb {R}^P$ (rotation scalings), optional trainable bias $\phi \in [-\pi ,\pi ]$.
- *Gateset.* $\{X, R_x, R_y, R_z, \textrm{CRY}\}$ (native-compilable on common backends).
- *Init/Meas.* $g:|0\rangle \xrightarrow {X}|1\rangle$, $t:|0\rangle$; measure *Z* on *t*.
- *Forward.* Deterministic map $s \mapsto y(s)$ given by Eq. (11) (up to additional rotations for $p>3$).
- *Backward.* Parameter-shift differentiation in Eq. (3) for each entry of $\boldsymbol{\theta }$.

This contract enables drop-in reuse, unit tests (e.g., verifying small-*s* gain matches Eq. (13)), and hardware co-design (e.g., mapping $\textrm{CRY}$ to native two-qubit gates while preserving bias semantics). It also allows *system-level* reasoning: operating-point selection, gain budgeting across layers, and robustness auditing under noise contractions.

In many sensing problems the positive class is rare, making *threshold choice* as important as score quality. Let $p_1(y)$ and $p_0(y)$ be the score densities for positive and negative classes on a validation fold, and let $\tau \in [-1,1]$ be a decision threshold on the QT (or network) score. The F1 score (harmonic mean of precision and recall) as a function of $\tau$ is

16 $$\begin{aligned} \textrm{F1}(\tau ) \;=\; \frac{2\,\textrm{TP}(\tau )}{2\,\textrm{TP}(\tau )+\textrm{FP}(\tau )+\textrm{FN}(\tau )}, \end{aligned}$$

with

17 $$\begin{aligned} \textrm{TP}(\tau )&= \pi _1\int _{\tau }^{1} p_1(y)\,dy, \quad \textrm{FP}(\tau ) = \pi _0\int _{\tau }^{1} p_0(y)\,dy, \end{aligned}$$

18 $$\begin{aligned} \textrm{FN}(\tau )&= \pi _1\int _{-1}^{\tau } p_1(y)\,dy,\qquad \textrm{TN}(\tau ) = \pi _0\int _{-1}^{\tau } p_0(y)\,dy, \end{aligned}$$

where $\pi _c$ are class priors on the validation fold. Maximizing $\textrm{F1}(\tau )$ yields a (typically unique) $\tau ^\star$ that is *not* generally 0.0 or 0.5; in our experiments we therefore *calibrate* $\tau$ on validation data and carry it unchanged to the held-out test split. From a functional-analytic viewpoint, any monotone contraction of scores (e.g., the noise factor $\lambda _Z$) leaves the maximizing $\tau ^\star$ *approximately invariant*, explaining the empirical stability of calibrated thresholds under moderate device drift.

Several quantum-learning abstractions echo neural primitives:

- *Quantum neurons / perceptrons.* A substantial body of work uses short parameterized circuits plus measurement to realize nonlinear scalar activations or perceptron-like decision rules; see, e.g.,^8,16^ and references therein. The QT block studied here should be viewed as a concrete instance of this general paradigm, specialized to a bias-last template with an explicit operating-point parameter.
- *Feature-map VQCs and data re-uploading.* It is well established that *s*-dependent rotations (and repeated “re-uploading” of inputs) generate expressive trigonometric feature maps in variational classifiers^8^. Our *p*-parameter QT is consistent with this view: increasing *p* enriches the harmonic content while keeping the block contract fixed.
- *Hardware-efficient templates and analyzable blocks.* Hardware-efficient VQCs typically trade global expressivity for shallow depth and improved trainability under realistic noise^2^. The QT contribution is at the *block level*: a reusable template whose input/output contract, gain/saturation characteristics, and compilation assumptions are explicit and can be tested and audited.

*Positioning and novelty.* In light of this prior art, we do not claim that “a few rotations + measurement” constitutes a fundamentally new quantum neuron. The incremental contributions of this work are (i) the transistor-inspired *small-signal* analysis (explicit *y*(*s*), $g_q(s)=\partial y/\partial s$, operating regions, and gain budgeting for stacked layers), (ii) an engineering-style *interface specification* for a reusable variational primitive (ports, initialization/measurement conventions, parameter-shift compatibility, and a compiler-facing bias interaction), and (iii) a system-level pipeline that treats calibration and monotone noise contraction as part of the block contract. We expanded this related-work discussion and added explicit citations to feature-map and re-uploading literature to make the relationship clear.

A practical architecture must balance expressivity against trainability and hardware constraints (qubit count, entangling depth, calibration complexity). The QT stack we study obeys three design guidelines:

1. *Shallow entanglement, local nonlinearity.* Each layer uses at most a single two-qubit biasing interaction within each QT and no inter-QT entanglers. In the experiments reported here the control qubit is always prepared in $|1\rangle$ before the bias, so $\textrm{CRY}(\phi )$ reduces to an unconditional $R_y(\phi )$ on the channel and, consequently, no multi-qubit entanglement is ever generated in the current prototype; the two-qubit interface and the entangling-budget figures we quote should therefore be read as an *upper bound* compatible with future variants in which *g* is genuinely data-dependent and may be shared across several channels. This keeps two-qubit error accumulation low and simplifies compilation.
2. *Linear contraction before quantum.* A classical linear map compresses high-dimensional inputs to a small set of normalized signals in $[-1,1]$. This acts as learned feature selection and *gain staging* that avoids early saturation.
3. *Per-layer gain budgeting.* Using Eq. (14) and Eq. (15), we set per-block scalings so that the product of layer Jacobian norms stays near unity in the first training epochs, allowing gradients to percolate without exploding or vanishing.

## Quantum transistor

A single QT consumes one real signal $s\in [-1,1]$ and returns one scalar $y(s)\in [-1,1]$ equal to the Pauli-*Z* expectation of a *channel* qubit *t* after interacting with a *control* qubit *g*. We initialize $g=|1\rangle$ (via a single *X*) and $t=|0\rangle$. The block is parameterized by $\boldsymbol{\theta }=(\theta _1,\ldots ,\theta _p)$ and a bias angle $\phi$ applied through a controlled $R_y(\phi )$ from *g* to *t*. To keep expressions compact we write $\kappa _j=\pi \theta _j$ and adopt the axis schedule $\alpha _1=y$, $\alpha _2=x$, $\alpha _3=z$; for $j\ge 4$ we use $\textrm{Rot}(\varphi )=R_z(\varphi )R_y(\varphi )R_x(\varphi )$ on *t*. The unitary and readout are

19 $$\begin{aligned} U(s;\boldsymbol{\theta },\phi ) =\textrm{CRY}^{(g\rightarrow t)}(\phi )\,\Bigg (\prod _{j=1}^{p} R_{\alpha _j}^{(t)}(\kappa _j s)\Bigg ), \quad y(s)=\langle 10|\,U^\dagger \,(I\otimes Z)\,U\,|10\rangle . \end{aligned}$$

One QT with $p=5$ parameters (three base rotations plus two data re-uploads) follows these semantics: all *s*-scaled single-qubit rotations act on the *channel* *t* first, the *control* *g* is prepared in $|1\rangle$ once via *X*, and the bias is applied *last* as a controlled $R_y(\phi )$.
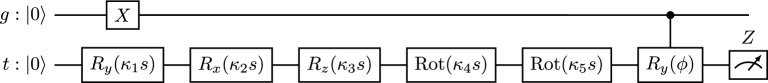


At the device level both physical wires of a QT are initialized in the ground state $|0\rangle$; the logical preparation $g=|1\rangle$, $t=|0\rangle$ in Eq. (19) is implemented by a single *X* gate on *g* at the beginning of the block. After the bias interaction and the *Z*-measurement on *t*, the joint state of the two wires factorizes as $|1\rangle _g\otimes \rho _t$, because in the present prototype the control is always deterministically in $|1\rangle$ when the $\textrm{CRY}(\phi )$ is applied and no other two-qubit gates couple *g* and *t*. The control line *g* is therefore *not* used as an input to any subsequent QT or classical post-processing in our experiments; it can be reinitialized or discarded between circuit evaluations without affecting the model. Operationally, this means that, for the specific instantiation studied here, the action of the bias gate is indistinguishable from an ordinary single-qubit $R_y(\phi )$ on *t* (as already reflected in the Bloch-sphere derivation leading to Eq. (11)), and the explicit control wire should be read as part of the *definition* of the standardized QT block and its future data-dependent generalizations, rather than as an additional source of entanglement in the present experiments.

*Reference implementation and compilation mapping.* The QT circuits used throughout this manuscript are provided as an executable PennyLane reference implementation available at https://zenodo.org/records/18559151, including the end-to-end gait pipeline and the exact parameter-shift gradient evaluations. In the specific instantiation evaluated in this manuscript, the control qubit is deterministically prepared as $|1\rangle$ and is not reused; under this constraint the controlled-$R_y(\phi )$ bias is compile-time equivalent to an unconditional single-qubit $R_y(\phi )$ on the channel and may be simplified by a compiler without changing the transfer function or gradients. We nevertheless describe the bias as a controlled rotation in the standardized template so that the same interface covers future data-dependent gating variants in which the control is nontrivial. At the gate level, when the controlled bias is implemented explicitly, on hardware with a native $\textrm{CRY}(\phi )$ the bias is a single two-qubit operation. When $\textrm{CRY}$ is not native, we compile the bias using the standard two-CNOT synthesis (consistent with the released PennyLane code and with backends exposing $\{R_x,R_z\}$ or related one-qubit natives).
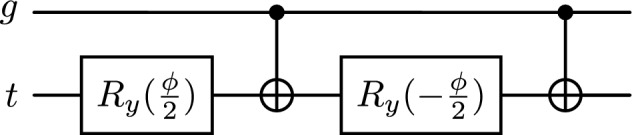
 which preserves the “bias-last” semantics. Each $R_y(\theta )$ can be compiled as $R_z(-\tfrac{\pi }{2})\,R_x(\theta )\,R_z(\tfrac{\pi }{2})$ on backends with $\{R_x,R_z\}$ natives. For $p=5$, the pre-bias stack contributes $3+3(p{-}3)=9$ *data-dependent* single-qubit rotations on *t*; the bias synthesis adds only *fixed* one-qubit rotations whose exact number depends on the native set. The entangling depth of one QT is constant in *p* (two CNOTs under the synthesis above) because all *s*-scaled rotations precede the bias and act locally on *t*. All performance numbers reported in this manuscript are obtained on simulators; we do not include cloud-QPU results here, and we treat hardware effects via the explicit gate-level mapping above together with the noise-contraction analysis in Sect. "Robustness to noise and calibration".

Every pre-bias angle is $\varphi _j(s)=\kappa _j s$. By the chain rule,

20 $$\begin{aligned} \frac{\partial y}{\partial s}=\sum _{j=1}^{p}\kappa _j\,\frac{\partial y}{\partial \varphi _j}. \end{aligned}$$

For generators with eigenvalues $\pm \tfrac{1}{2}$, the parameter-shift rule gives exact derivatives without stochastic backpropagation through measurement:

21 $$\begin{aligned} \frac{\partial y}{\partial \varphi _j}&=\tfrac{1}{2}\Big [y\big |_{\varphi _j+\frac{\pi }{2}}-y\big |_{\varphi _j-\frac{\pi }{2}}\Big ], \frac{\partial y}{\partial \theta _j} =\pi s\cdot \tfrac{1}{2}\Big [y\big |_{\varphi _j+\frac{\pi }{2}}-y\big |_{\varphi _j-\frac{\pi }{2}}\Big ], \frac{\partial y}{\partial \phi } =\tfrac{1}{2}\Big [y\big |_{\phi +\frac{\pi }{2}}-y\big |_{\phi -\frac{\pi }{2}}\Big ]. \end{aligned}$$

A single backward step over one QT therefore requires 2*p* shifted evaluations for $\boldsymbol{\theta }$; these are depth-constant circuits that reuse the same readout.

The classical front-end is deliberately minimal. Each spectrogram window is flattened to a vector $x\in \mathbb {R}^{D}$, and a single fully connected layer $W\in \mathbb {R}^{8\times D}$ with bias $b\in \mathbb {R}^8$ followed by a pointwise $\tanh$ produces eight bounded signals,

22 $$\begin{aligned} h=\tanh (Wx+b)\in [-1,1]^8. \end{aligned}$$

In the gait experiments $D=40\cdot 12=480$, so this “classical front-end” is simply one affine map (3, 840 weights and 8 biases, 3, 848 parameters in total) and no additional classical hidden layers. Its parameters are trained jointly with the QT parameters by back-propagation; there is no separate deep classical encoder in front of the quantum stack.

Write $h=(h_1,\dots ,h_8)$. In the 4–3–2 QT layout used throughout the experiments, the three quantum layers consume these coordinates as follows:

- *Layer 1 (4 QTs, 8 qubits).* The four QTs in Layer 1 take as inputs the first four contracted features, i.e., their scalar inputs are
  $$(s^{(1)}_1,\dots ,s^{(1)}_4)=(h_1,h_2,h_3,h_4),$$
  and they output $z^{(1)}=(z^{(1)}_1,\dots ,z^{(1)}_4)\in [-1,1]^4$. The remaining contracted coordinates $h_5,\dots ,h_8$ are not wired into this particular QT layout and therefore do not influence the classifier; they can be regarded as unused slack dimensions of the contraction layer for this architecture.
- *Layer 2 (3 QTs, 6 qubits).* Layer 2 receives only the first three outputs of Layer 1: its QTs take
  $$(s^{(2)}_1,s^{(2)}_2,s^{(2)}_3)=(z^{(1)}_1,z^{(1)}_2,z^{(1)}_3)$$
  and produce $z^{(2)}=(z^{(2)}_1,z^{(2)}_2,z^{(2)}_3)\in [-1,1]^3$. The fourth output $z^{(1)}_4$ is not forwarded to deeper layers and does not enter the loss; we retain it only as an auxiliary diagnostic channel (it appears, together with the other components of $z^{(1)}$, in the per-layer $\langle Z\rangle$ trajectories plotted in the Results section).
- *Layer 3 (2 QTs, 4 qubits).* Layer 3 takes as inputs the first two outputs of Layer 2,
  $$(s^{(3)}_1,s^{(3)}_2)=(z^{(2)}_1,z^{(2)}_2),$$
  and returns $z^{(3)}=(z^{(3)}_1,z^{(3)}_2)\in [-1,1]^2$. The second component
  $$\ell \equiv z^{(3)}_2$$
  is used as the logit for the binary classifier, with $\hat{p}=\sigma (\ell )$ the predicted probability and a validation-calibrated threshold applied to $\hat{p}$ at test time. The first component $z^{(3)}_1$, like $z^{(1)}_4$ and $z^{(2)}_3$, is not consumed by any downstream layer or the loss and serves only as an auxiliary output that we log for completeness.

*Index-preserving signal flow and logit dependence.* In the present 4–3–2 instantiation, each QT consumes a single scalar and there is no inter-QT mixing (no fan-in) within or across layers. We therefore use an *index-preserving* wiring rule: for any layer transition, QT *i* in the next layer takes as input the output of QT *i* in the previous layer (for indices that exist in both layers), i.e.,

$$s^{(\ell +1)}_i \;=\; z^{(\ell )}_i,\quad i=1,\dots ,\min (n_\ell ,n_{\ell +1}).$$

In particular, for the reported 4–3–2 layout we have $s^{(2)}_i=z^{(1)}_i$ for $i=1,2,3$ and $s^{(3)}_i=z^{(2)}_i$ for $i=1,2$. Because the logit is defined as $\ell \equiv z^{(3)}_2$, the classifier depends only on the single chain

$$x \;\longrightarrow \; h_2 \;\longrightarrow \; z^{(1)}_2 \;\longrightarrow \; z^{(2)}_2 \;\longrightarrow \; z^{(3)}_2.$$

Consequently, although we execute (and log) the full 4–3–2 template, the *effective gradient-carrying subgraph* for the reported experiments is functionally equivalent to a 1–1–1 chain. All other quantities—including $h_1,h_3,h_4,\ldots ,h_8$ and $z^{(1)}_1,z^{(1)}_3,z^{(1)}_4,z^{(2)}_1,z^{(2)}_3,z^{(3)}_1$—do not enter $\ell$ and therefore have identically zero gradient with respect to the loss; they can be pruned without changing the predictions. We retain the wider template for diagnostic logging and to keep a fixed scaffold for future multihead/pooling variants in which multiple chains are explicitly combined into the final logit.

To avoid ambiguity, we distinguish two notions throughout this manuscript. The *executed template* is the full 4–3–2 scaffold that is evaluated and logged (nine QTs plus the 8-output contraction layer). The *effective trainable model* is the subset of operations and parameters that actually influence the score used by the loss and therefore receive nonzero gradient. Under the index-preserving wiring and the single-head logit $\ell =z^{(3)}_2$, only one contraction coordinate and one QT per layer lie on the decision path. Consequently, the executed template contains 9*p* declared QT parameters and $3\,848$ contraction parameters (total $3\,848+9p$, i.e., $3\,893$ at $p=5$), whereas the effective trainable model contains only 3*p* QT parameters and $480+1=481$ contraction parameters (total $481+3p$, i.e., 496 at $p=5$). The same distinction applies to compute: the present code evaluates all nine QTs (thus 18*p* parameter-shift circuit evaluations per batch), while a pruned implementation that evaluates only the effective single chain would require 6*p* shifted evaluations per batch with identical predictions. We retain the wider executed template for diagnostic logging and as a fixed scaffold for planned multi-output readouts, but we interpret capacity claims in terms of the effective trainable model described here.

**Fig. 1:**
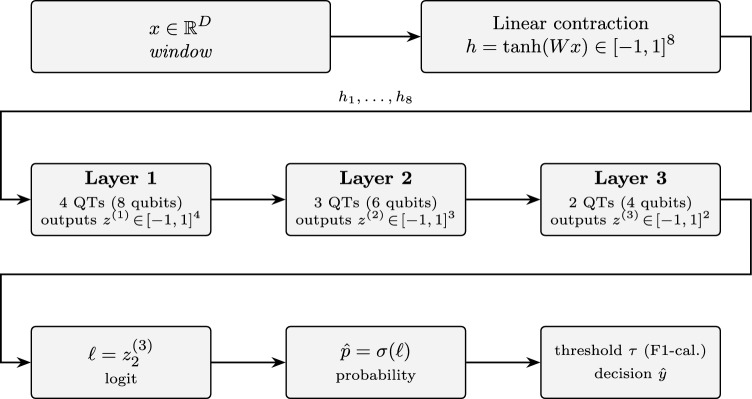
End-to-end pipeline (vertical layout): linear contraction (Eq. (22)) produces eight bounded signals; three QT layers process them without inter-QT entanglement; the second output of the last layer is the logit ($\ell =z^{(3)}_2$), followed by a sigmoid and a validation-calibrated threshold. Each QT implements the block of Sect. "Quantum transistor" with $p=5$ (three base rotations plus two re-uploads) and a single bias interaction $\textrm{CRY}(\phi )$. In the 4–3–2 layout only $h_1,\dots ,h_4$ are evaluated by Layer 1, $(z^{(1)}_1,z^{(1)}_2,z^{(1)}_3)$ are evaluated by Layer 2, and $(z^{(2)}_1,z^{(2)}_2)$ are evaluated by Layer 3. However, because the wiring is index-preserving ($s^{(\ell +1)}_i=z^{(\ell )}_i$) and the logit is defined as $\ell =z^{(3)}_2$, the classification decision depends only on the single chain $x\!\rightarrow \!h_2\!\rightarrow \!z^{(1)}_2\!\rightarrow \!z^{(2)}_2\!\rightarrow \!z^{(3)}_2$; all other intermediate outputs are diagnostic and do not affect the decision.

Figure 1 provides an overview of the end-to-end hybrid pipeline, highlighting the classical contraction (Eq. (22)), the three QT layers, and the validation-calibrated thresholding step used for held-out evaluation.

### Robustness to noise and calibration

Single-qubit noise on the measured channel *t* is conveniently analyzed in the PTM picture. Any completely positive trace-preserving map $\mathcal {E}$ on a single qubit acts affinely on $(1,\textbf{v})$ as $(1,\textbf{v})\mapsto (1,\textbf{t}+T\textbf{v})$, where $\textbf{v}$ is the Bloch vector and $T\in \mathbb {R}^{3\times 3}$. For trace-preserving, unital noise ($\textbf{t}=\textbf{0}$) with a diagonal $T=\textrm{diag}(\lambda _X,\lambda _Y,\lambda _Z)$, the QT output contracts as

23 $$\begin{aligned} y_{\textrm{noisy}}(s)=\lambda _Z\,y_{\textrm{ideal}}(s), \,\,\, \frac{\partial }{\partial s}y_{\textrm{noisy}}(s)=\lambda _Z\,\frac{\partial }{\partial s}y_{\textrm{ideal}}(s). \end{aligned}$$

Depolarizing noise with rate *p* yields $\lambda _Z=1-\tfrac{4}{3}p$; pure dephasing about *Z* leaves $\lambda _Z=1$ (since *Z* is an eigenoperator). Thus, under common unital channels, the transfer curve’s shape and the location of its saturation edges are preserved, with a vertical contraction that scales both output and slope.

Non-unital channels add a bias term. For example, amplitude damping with small rate *p* maps $Z\mapsto \lambda _Z Z + t_Z I$ with $\lambda _Z\approx 1-p$ and $t_Z\approx p$, so

24 $$\begin{aligned} y_{\textrm{noisy}}(s)\approx \lambda _Z\,y_{\textrm{ideal}}(s)+t_Z, \end{aligned}$$

which shifts the operating point while contracting the slope. In our block, such a shift can be counteracted by adjusting the bias angle $\phi$ (or by recentring outputs before thresholding) without modifying the small-slope control given by $\partial y/\partial s$ in Eq. (23). Readout bit-flip error with probability $p_r$ has the simple effect $y_{\textrm{noisy}}=(1-2p_r)\,y_{\textrm{ideal}}$, a purely multiplicative factor that can be calibrated with standard readout-mitigation routines. Two-qubit errors accrued during the $\textrm{CRY}$ (or its two-CNOT synthesis) further contract |*y*|; to first order they act like an additional multiplicative factor $\lambda _{\textrm{2q}}\in (0,1)$ on the effective slope. In all these cases the multiplicative structure maintains the ordering of scores and hence keeps a validation-calibrated decision threshold $\tau$ approximately invariant under moderate drift, which matches our empirical stability across folds.

Training minimizes a class-weighted logistic loss on the logit $\ell$:

25 $$\begin{aligned} \mathcal {L}(\ell ,y;\lambda )=\lambda \,y\,\log \!\big (1+e^{-\ell }\big )+(1-y)\,\log \!\big (1+e^{\ell }\big ), \quad \lambda =\texttt {pos\_weight}. \end{aligned}$$

With $\sigma (\ell )=1/(1+e^{-\ell })$, the gradient wrt. $\ell$ is

26 $$\begin{aligned} \frac{\partial \mathcal {L}}{\partial \ell }= \sigma (\ell )\big (1+(\lambda -1)y\big )-\lambda \,y \;=\; (\sigma (\ell )-y)\;-\;(\lambda -1)\,y\,(1-\sigma (\ell )), \end{aligned}$$

so positives are up-weighted by $\lambda$ and negatives by 1, which matches the implementation of BCEWithLogitsLoss used in our code. We select the decision threshold $\tau$ on each validation fold to maximize F1 and carry that $\tau$ unchanged to the corresponding test fold; this aligns the operating point with the task’s utility under class imbalance and is robust to the monotone contractions described above.

One forward pass uses at most eight concurrent qubits (Layer 1), and the bias interaction contributes either zero two-qubit gates (if the compiler collapses the controlled rotation under the fixed-control constraint of the present experiments) or, as a conservative upper bound for the standardized two-wire template, $2\times (4+3+2)=18$ two-qubit gates if the controlled bias is implemented explicitly and layers are executed sequentially, and 9 data-dependent single-qubit rotations per QT when $p=5$. Exact gradients via the parameter-shift rule require 2*p* shifted circuits per QT for the parameters $\boldsymbol{\theta }$ (and +2 if $\phi$ is trainable). With nine QTs and $p=5$, one optimizer step therefore evaluates 90 shifted circuits plus one unshifted forward (for the loss), all at constant entangling depth. If *M* shots are used per circuit to estimate $\langle Z\rangle$, the per-update shot budget is $(90+1)\times M$, which provides a transparent accounting for both simulation and hardware execution.

The axis schedule for the first three parameters is $(R_y,R_x,R_z)$ with angles $(\kappa _1 s,\kappa _2 s,\kappa _3 s)$, which ensures a clean small-signal slope controlled by $R_y$ and the bias $\phi$. Parameters $j\ge 4$ each add a data re-upload $\textrm{Rot}(\kappa _j s)\equiv R_z(\kappa _j s)R_y(\kappa _j s)R_x(\kappa _j s)$ on the channel, enriching the harmonic content while keeping the first-order slope governed by the effective *y*-axis coefficient. The bias is fixed to $\phi =\pi /3$ in reported runs to guarantee a nonzero and sizable small-signal slope via

27 $$\begin{aligned} \left. \frac{\partial y}{\partial s}\right| _{s=0}=-\kappa _1\sin \phi , \end{aligned}$$

making $\phi$ trainable is straightforward via the same parameter-shift rule used for $\boldsymbol{\theta }$ (see Eq. (21)). Unit tests validate three aspects: (i) the closed-form transfer against a statevector simulator for random settings, (ii) the small-*s* slope in Eq. (27) via finite differences at $s\approx 0$, and (iii) parameter-shift gradients in Eq. (21) against automatic differentiation. This test suite guards against drift between the analytic specification and the executable pipeline, and it supports portable compilation to backends with native $\textrm{CRY}$ or its two-CNOT synthesis without altering the block’s bias-last semantics.

From a simulation perspective, the QT block is particularly amenable to GPU acceleration. Each forward evaluation of Eq. (11) and its derivatives in Eqs. (20)–(21) reduces to a small, fixed sequence of trigonometric operations on the scalars $(s,\boldsymbol{\theta },\phi )$. A stacked QT network can therefore be implemented as batched tensor operations in standard automatic-differentiation frameworks that already support GPU and TPU backends. In such an “analytic” implementation one does not materialize an *n*-qubit state vector; instead, the simulator evaluates the closed-form Bloch-sphere expressions per block and per data point, which scale linearly with the number of QTs and the batch size and are straightforward to fuse into GPU kernels. This makes the QT definition reliable both as a hardware primitive and as a building block for large-scale, GPU-accelerated classical simulations of deeper QT networks.

Because, on hardware, each QT score is obtained as the empirical mean of *M* bounded $\{\pm 1\}$ outcomes, its variance decays as $O(M^{-1})$ and the induced fluctuations in F1 and in the validation-selected threshold follow the standard $O(M^{-1/2})$ concentration behaviour of Monte Carlo estimators rather than any QT-specific pathology. In this sense, the statevector results reported here correspond to the high-shot limit, and the PTM-based monotone contraction described above characterizes how realistic noise channels would smoothly deform the corresponding F1–versus–shots curves and threshold location without introducing unexpected instabilities.

In summary, the QT implements a bounded, transistor-like transfer $s\mapsto y(s)$ with analytic control of operating point and gain, exact gradients via parameter shift, and a constant, low entangling cost. Stacking QTs under the gain budget in Eq. (15) yields a shallow network that integrates cleanly with a classical front-end and a logistic head while remaining compatible with near-term hardware.

## Classical baselines and data collection process

**Fig. 2:**
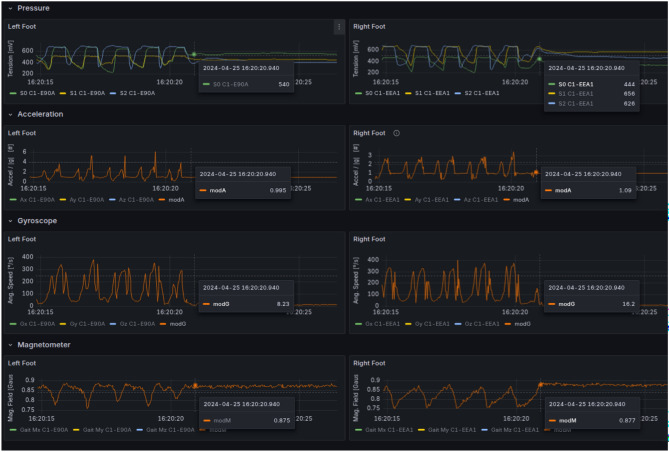
Data collected from Sesonria Inc trademark socks. Pressure but also acceleration, rotations from gyroscopes and magnetometer signals have been recorded in an InfluxDB engine.

To validate the capabilities of the proposed approach, an application is developed in real life. The main interest is to be able to identify gait when the collected data come from instrumented socks sensors, in such a way that both legs show the appropriate pattern (see Fig. 2).

Motivation is driven by the clinical need to detect motor alterations in their earliest stages, even before they become apparent to a neurologist during a standard examination. By treating human gait as a rich and accessible “digital biomarker,” the project aims to build a system that captures the nuances of a patient’s movement in their real-world environment, transforming a routine activity into a powerful source of clinical insight.

The rigorous approach adopted, guided by the CRISP-DM framework and validated with metrics like F1 score, ensures that the final model is not only effective but also robust and well-justified.

The process begins by targeting specific time intervals of interest, and for each interval, the script queries a time series database (InfluxDB) to retrieve raw sensor data for the left and right feet. This raw data includes multiple channels, such as Pressure (three points per foot), Accelerometer, and Gyroscope modules. A critical data preparation step is synchronization and resampling. Since sensor readings from two separate devices (one for each foot) are not perfectly aligned, the pipeline first identifies the common, overlapping time window for both feet. It then creates a new, perfectly uniform time grid at a target frequency (e.g., 70 Hz). The raw data from both feet are then interpolated onto this common time base, resulting in two perfectly synchronized, clean, and consistently sampled time series DataFrames. This step is essential for any meaningful comparative analysis.

Rather than using the raw time series data directly, the pipeline transforms the signals into a frequency-domain representation using Power Spectral Density (PSD) spectrograms (five key signals per foot). This spectrogram computation is the core of the feature-extraction stage: it captures how the signal’s frequency content evolves over time and is therefore well suited to the rhythmic structure of walking. A spectrogram reveals how the frequency content of a signal evolves over time, making it excellent for identifying the rhythmic patterns inherent in walking. The resulting PSD values are converted to a decibel (dB) scale, normalized, and scaled to an 8-bit integer range (0–255), effectively turning each signal into a grayscale image. By this way features are less sensitive to the absolute values of the signals, in particular because of the long-term decay and drift of the pressure sensors. Finally, the five individual spectrograms for each foot are stacked vertically to create a single 2D feature tensor, or “image,” representing the complete sensor profile for that foot over the time interval. If data from both feet were successfully processed, their respective tensors were also stacked together to form a comprehensive dual-foot tensor. The pipeline then converts raw, asynchronous sensor readings into clean, synchronized, and highly informative 2D feature tensors that encode the rich frequency characteristics of a subject’s gait over time.

Our analysis will start from the features described earlier, and to ensure a robust and unbiased evaluation, a grouped K-fold cross-validation by subject/session is used. For consistency with the QT pipeline, all classical models receive as input exactly the same spectrogram windows that are stored as $40\times 12$ grayscale images in the processed_images dataset. In the quantum case these images are flattened and mapped linearly to $h=\tanh (Wx)\in [-1,1]^8$ by the contraction layer in Eq. (22) before entering the QT stack. In the classical baselines, the CNN and Transformer operate directly on the full $40\times 12$ tensors without this explicit contraction stage. Thus the classical models have access to a strictly richer representation than the QT block interface, and the reported F1 gap between them should be read as a conservative upper bound on the gap that would remain if the classical models were also restricted to the same 8-D contracted features. We benchmark the Quantum Transistor approach against more traditional artificial intelligence baselines:

- Compact 2D CNN on spectrogram-like inputs;
- Transformer encoder that treats time frames as tokens and frequency bins as features;
- CMMA placeholder as a simplified multi-scale attention design;
- A logistic-regression classifier on flattened spectrogram windows (approximately $4.8\times 10^2$ trainable parameters);
- A one-hidden-layer “tiny” MLP with 8 hidden units on the same flattened inputs (approximately $3.86\times 10^3$ trainable parameters), chosen to closely match the QT network’s *executed-template* trainable parameter count.

All baselines use the same cross-validation protocol, early stoppage, and learning rate reductions.

The lightweight logistic and MLP baselines are configured deliberately to probe the “compactness” axis raised in the review. Both operate directly on the same $40\times 12$ spectrogram windows as the CNN and Transformer, flattened into $D=480$-dimensional vectors. The logistic model has $D+1=481$ trainable parameters, i.e., 0.124 times the QT network’s executed-template total of $3893$ parameters. However, because the present QT instantiation uses an index-preserving wiring and a single-head readout, its effective gradient-carrying computation graph has $481+3p$ trainable parameters; at $p=5$ this is 496 parameters, so the logistic model is 0.97 times the effective trainable size. The tiny MLP uses a single hidden layer with 8 ReLU units and a scalar output, for a total of $3857$ trainable parameters, i.e., 0.991 times the QT network’s executed-template parameter budget ($3893$ parameters including the classical contraction). Throughout the paper we therefore distinguish the executed template (all blocks evaluated and logged, which sets runtime and hardware budgets) from the effective trainable model (the subset that actually influences the score used by the loss in this single-head prototype).

From the perspective of model capacity, the classical front-end used by the QT network is thus a single shallow linear map plus $\tanh$ on top of the raw $40\times 12$ spectrogram windows, with $3848$ trainable parameters in the executed template. In the present single-head, index-preserving instantiation only one of the eight contracted coordinates lies on the decision path, so only 480 weights plus one bias (481 parameters) receive nonzero gradient. In contrast, the CNN and Transformer baselines operate directly on the same spectrogram tensors but include multiple learned convolutional or attention layers with significantly larger parameter budgets (on the order of $10^5$ parameters for the CNN under its best HyperBand configuration). In other words, in the QT pipeline the classical component before the quantum stack is intentionally kept low-capacity and serves primarily as a compact, differentiable “read-out” of the spectrogram into eight bounded features; we do not insert a deep classical feature extractor in front of the QT layers. This design choice reflects the focus of the present work on the behaviour of the standardized QT block under a simple, transparent classical pre-processing stage.

### Data collection

The data collection application was created for both Android and iOS platform in such a way that it can be installed on the patient’s smartphone. The device pairing process is intentionally simplified. Instead of a complex multistep procedure, the user simply taps a button in the app for the desired device (e.g., “Left Sock”) and brings the phone close to it. The app automatically detects and connects to the nearest device by selecting the one with the highest Received Signal Strength Indicator (RSSI).

Before the session begins, the sensors are calibrated to ensure data accuracy. The smart socks are zeroed while the patient is seated, and the motion sensors are initialized to ensure that their orientation readings are consistent. Once all devices are connected and calibrated, their status is clearly displayed on the app’s main dashboard.

While the person is moving, the data capture app works automatically to capture and manage the data flow, by collecting high-frequency (50–100 Hz) data streams in sync from all connected sensors. To prevent data loss due to network interruptions, the app employs a robust buffering mechanism. Incoming data are timestamped, buffered locally, compressed, and then periodically (e.g., every 60 seconds) uploaded to the cloud server via a secure HTTPS protocol. The local data file is only deleted after the server confirms that it has been successfully received, ensuring data integrity.

The data set was collected from human subjects using instrumented socks providing pressure and inertial sensing. To avoid repetition, the preprocessing and feature-extraction steps (synchronization/resampling and PSD spectrogram windows) follow the pipeline described in Sect. "Classical baselines and data collection process"; here we focus on the collection workflow and the automatic labeling strategy used to identify sustained walking events.

An automated labeling engine was implemented to identify true walking events. This algorithm uses multi-sensor fusion and Short-Time Fourier Transform (STFT) to detect the characteristic periodic energy signatures of human gait (typically 0.6–2 Hz). To ensure clinical relevance, only sustained walking periods lasting more than five seconds were labeled as “walking,” thus filtering out minor incidental movements. This process transformed the unlabeled real-world data into a structured data set suitable for training and validating machine learning models.

This robust identification of gait periods is profoundly meaningful because it unlocks the ability to generate the “rich semantic higher-level description”. The automatic gait identification process serves as a powerful labeling engine. It looks to transform hours of unlabeled, real-world data into a structured data set where every segment is tagged as “walking” or “not-walking.” This consistent, labeled dataset is a “crucial foundation” for training and validating more advanced AI models.

### Best practices and ethics

All data collection involving human subjects was carried out under rigorous ethical governance, receiving formal approval from the Ethics Committee of Getafe University Hospital (CEIm) and the Technical Committee of the Universidad Politécnica de Madrid. All methods were performed in accordance with the relevant guidelines and regulations. The system was designed with privacy as a core principle. A secure and anonymized reference code system was used, ensuring that no personally identifiable information was stored on the mobile device. Informed consent was obtained from all subjects. All data were encrypted during transmission using the HTTPS protocol to protect the confidentiality of the participants. The evaluation protocol ensures subject-grouped splits for cross-validation, full reproducibility, and no threshold selection on the test set, in adherence with best practices.

The system was architected with privacy as a core component, implementing several mechanisms to protect sensitive health information. A key feature is the use of a secure and privacy-preserving reference code system. Instead of using personally identifiable information, such as names or contact details, clinicians generate a unique anonymized reference code for each patient through a secure Web portal. This token is the only identifier used by the app and is explicitly designed to avoid storing any personal details on the mobile device. The collected datasets are described using non-personal identifiers such as timestamps and the MAC addresses of the wearable devices. The critical link between these anonymized data and the actual identity of the patient is maintained exclusively within the secure protocols of the healthcare system and never leaves that protected perimeter.

All data transmitted from the mobile application to the back-end server is sent using the HTTPS protocol, which encrypts the information to protect it from interception or unauthorized access. From this layer it is possible to ensure (i) subject-grouped splits, (ii) full reproducibility via fixed seeds and explicit config logs, (iii) no threshold selection on the test set, and (iv) export of per-epoch curves and confusion matrices. As gait can be considered biometric, we emphasize privacy-preserving handling and restrict claims to activity-state classification.

## Results and analysis

As a first step, we ablated network *depth and fan-in* while keeping the per-block semantics, gateset, and bias-last layout fixed (Sect. "Quantum transistor"). Because the present prototype uses an index-preserving wiring and a single-head readout, only one propagated chain contributes to the score and receives gradient signal; this sweep should therefore be read primarily as an executed-template runtime/resource comparison, not as a study of multi-path learning across the full template. We compared three stacks under the same subject-grouped protocol with $p=5$ pre-bias rotation parameters per QT: a deep 8–4–2–1 layout (15 QTs; max. concurrent qubits $=16$; entangling budget $2\times (8+4+2+1)=30$ per sequential forward), the intermediate 4–3–2 layout used elsewhere (9 QTs; max. concurrent qubits $=8$; entangling budget 18), and a shallow 2–1 layout (3 QTs; max. concurrent qubits $=4$; entangling budget 6). The deep 8–4–2–1 model proved *computationally impractical* and poorly conditioned in early training: epoch wall-times escalated to $t\!\approx \!3.78\times 10^{4}\,\textrm{s}$ (epoch 1) and $t\!\in \![0.81,1.40]\times 10^{4}\,\textrm{s}$ (epochs 2–4), while validation remained near chance (e.g., va$\approx 0.279$, F1$\approx 0.437$ at a threshold 0.01 across the first four epochs), so we discontinued this configuration. The shallow 2–1 model trained quickly but *underfit*, reaching mean test accuracy 0.954 and mean test F1 0.918 (fold-wise F1 $[0.930,\,0.909,\,0.914]$). In contrast, the intermediate 4–3–2 stack offered the best accuracy-vs-compute trade-off and stable optimization (Sect. "Quantum transistor"); we therefore adopt it for the subsequent HyperBand search over learning rate $\eta$ and per-block parameter count *p*.

HyperBand explored the learning rate $\eta$ and the per-block parameter count $p\in \{2,3,4,5\}$. The best configuration consistently selected $(\eta ^\star , p^\star )=(4.318\times 10^{-4},\,5)$, i.e., the QT with three base rotations plus two data re-uploads (Sect. "Quantum transistor"). During the bandit phase, trials separated early into two regimes: (i) underfitting runs with flat validation curves, and (ii) well-conditioned runs which crossed validation F1 $\approx 0.90$ by epoch 6 and then improved steadily without oscillations. This pattern is consistent with the gain-budgeted design of the stack: with $\phi =\pi /3$ and modest initial $\boldsymbol{\theta }$, the product of layer-wise Jacobian norms stays near unity, enabling gradients to percolate and keeping the model in the mid-slope region of the QT transfer. This partially answers *RQ4*: within a block, increasing the number of pre-bias rotations to $p=5$ (two re-uploads) improved validation F1 over $p\in \{2,3,4\}$ while preserving the block’s constant entangling cost.

Within the fixed 4–3–2 layout (4 QTs in Layer 1, 3 in Layer 2, 2 in Layer 3), the number of per-block re-uploads *p* controls expressivity while leaving the entangling depth per block constant. Each QT has *p* rotation scalings on its channel qubit, so the three layers contain 4*p*, 3*p*, and 2*p* QT parameters respectively, for a total of 9*p* trainable quantum parameters. The classical front-end contraction $h=\tanh (Wx)$ in Eq. (22) contributes $8\times (40\cdot 12)+8=3848$ additional parameters, independent of *p*, so the total parameter count of the whole QT network is $3848+9p$. Training-time cost scales linearly in *p* because parameter-shift differentiation requires 2*p* shifted circuits per QT (Eq. (21)), i.e., 18*p* shifted circuits plus one unshifted forward per batch for the 4–3–2 stack, while the number of two-qubit bias interactions (one per QT; two CNOTs when $\textrm{CRY}$ is decomposed) remains unchanged.

Table 2 summarizes the corresponding parameter counts and the HyperBand-observed validation $F_1$ ranges for each re-upload setting *p*.

**Table 2:** Expressivity–cost summary for the number of re-uploads *p* in the 4–3–2 QT stack. “QT params” counts only block-local rotation scalings; “total params” adds the $3848$ parameters of the classical contraction layer. These counts refer to the executed template (all nine QTs), which governs runtime and hardware budgeting. Under the present index-preserving single-head readout, the effective gradient-carrying model reduces to a 1–1–1 chain and therefore has $481+3p$ effective trainable parameters (496 at $p=5$). “HyperBand best $F_1$” reports the range of best validation F1 over the HyperBand trials at each *p* (logs available in the released code).

*p*	QT params (L1/L2/L3; total)	Total trainable params	# trials	HyperBand best $F_1$ (min–max)
2	$8/6/4;\;18$	$3866$	3	0.70–0.99
3	$12/9/6;\;27$	$3875$	3	0.99–1.00
4	$16/12/8;\;36$	$3884$	1	0.89
5	$20/15/10;\;45$	$3893$	3	0.99–1.00

Across the ten HyperBand trials ($p\in \{2,3,4,5\}$), all configurations with $p\ge 3$ reached best validation F1 $\ge 0.99$, while the $p=2$ setting showed one unstable run (best validation F1 $\approx 0.70$) and two high-performing runs (best validation F1 $\approx 0.99$). The densest block, $p=5$, thus offers the most consistently strong validation performance at essentially the same parameter budget: increasing *p* from 2 to 5 raises the total number of trainable parameters only from $3866$ to $3\,893$ (a $\approx 0.7\%$ increase), while the number of trainable quantum parameters grows from 18 to 45. Because HyperBand co-optimizes *p* and the learning rate and uses early stopping, this sweep is not a perfectly controlled grid over *p*, but it does show that larger *p* improves stability of high-F1 solutions without materially changing the parameter count or entangling depth; we therefore fix $p=5$ in the main experiments.

**Fig. 3:**
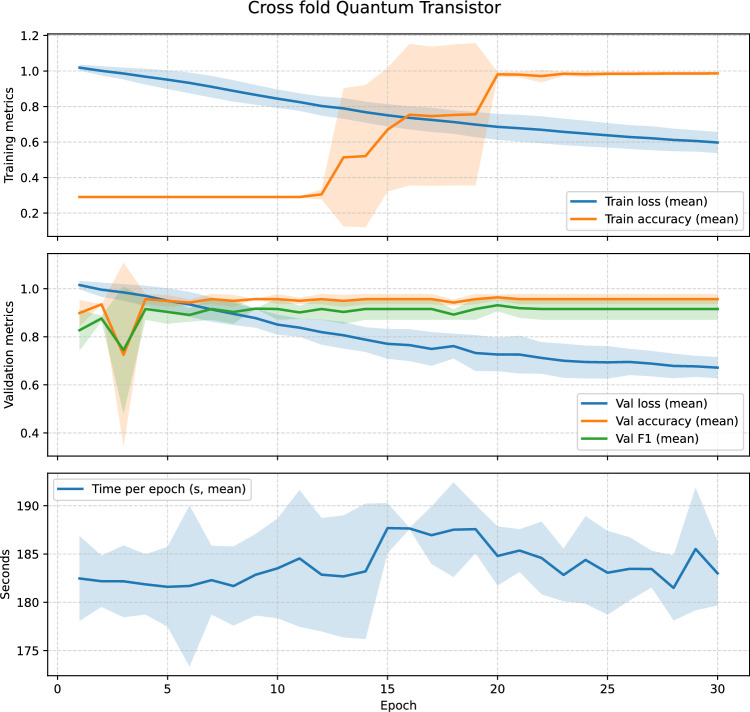
Cross-fold training (top), validation (middle), and wall-clock time (bottom) vs. epoch. Curves show the mean across the three folds with shaded ±1 s.d. bands. The switch-like rise in training accuracy after $\sim$epoch 12 is consistent with the QT’s transistor-like move from saturation toward the mid-slope gain region.

We summarize the cross-fold training dynamics in Fig. 3. Each curve is the mean over the three folds with a shaded ±1 s.d. band; the top panel shows training loss and accuracy, the middle panel validation loss/accuracy/F1, and the bottom panel wall-clock time per epoch. Two features stand out. First, while the training loss decreases smoothly from $\approx 1.03$ to $\approx 0.56$, the training accuracy remains near $\sim 0.29$ through roughly epoch 12 and then undergoes a switch-like transition to $\approx 0.93$–0.98. This “turn-on” matches the intended transistor-like behavior: as the QTs move their operating points off saturation and into the mid-slope gain region, the effective transconductance increases and the channel “opens,” yielding an abrupt rise in accuracy (consistent with the small-signal model in Eq. (27)). Second, validation metrics improve earlier and stabilize (F1 surpasses $\sim 0.90$ within the first few epochs), indicating that the operating-point shift primarily manifests in the training accuracy curve. Epoch times remain nearly constant at $\sim 180$–190 s with low fold-to-fold variance.

From epoch 5 onward the validation accuracy curve in Fig. 3 appears almost flat while the training accuracy continues to increase. This behaviour is expected in our setting. First, the plot shows the *mean* over subject-grouped folds; each fold reaches its own saturation point after a small number of epochs, and averaging these slightly misaligned saturation times yields an apparently plateau-shaped curve. Second, once the model has entered a high-accuracy regime on the relatively small validation sets, further decreases in the (class-weighted) logistic loss mostly sharpen the margins on examples that are already correctly classified, so training accuracy can still improve while validation accuracy has effectively saturated. The transient drop in validation accuracy around epoch 3 is due to the discrete nature of the 0/1 metric under class imbalance: early in training, small shifts in the predicted scores can move a handful of borderline windows across the decision boundary, which changes the validation accuracy more abruptly than the underlying probabilistic loss.

**Fig. 4:**
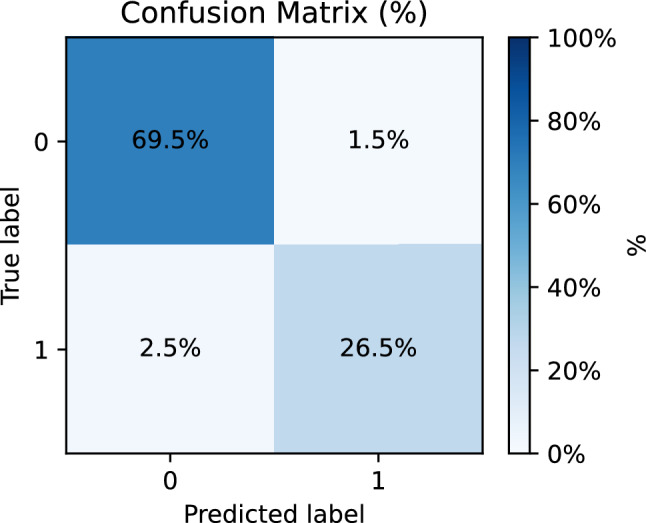
Cross-fold mean confusion matrix (percent of total). Grouped validation selects a per-fold threshold $\tau$ to maximize F1; the same $\tau$ is used on the held-out test fold.

**Fig. 5:**
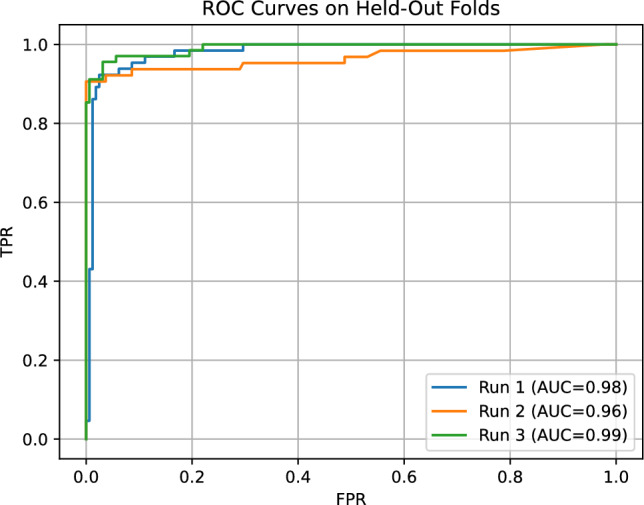
ROC curves on held-out folds for the QT network. Shaded variability reflects fold-to-fold differences under subject-grouped splits.

Using $(\eta ^\star ,p^\star )$ in a fresh, subject-grouped 3-fold evaluation, we obtained test accuracy per fold = [0.9427, 0.9735, 0.9648], mean = 0.960, $\text {stdev}\approx 0.0159,$ test F1 per fold = [0.9023, 0.9508, 0.9394], mean = 0.931, $\text {stdev}\approx 0.0254.$ Averaging the test-fold confusion matrices yields approximately $\textrm{TN}=157.7$, $\textrm{FP}=3.3$, $\textrm{FN}=5.7$, $\textrm{TP}=60.0$ per fold (fold size $\approx 227$), which aggregates to micro-averaged precision $\approx 0.948$, recall $\approx 0.913$, and F1 $\approx 0.930$. These values reflect a threshold calibrated on each validation fold to maximize F1 and then held fixed for its corresponding test fold (see Sect."Quantum transistor"). Figure 4 shows the cross-fold mean confusion matrix in percentage form; Figure 5 reports ROC curves over held-out folds. The operating points chosen by validation calibration sit on the high-precision, high-recall shoulder of the curves, matching the observed $\textrm{FP}\!\ll \!\textrm{FN}$ asymmetry.

**Fig. 6:**
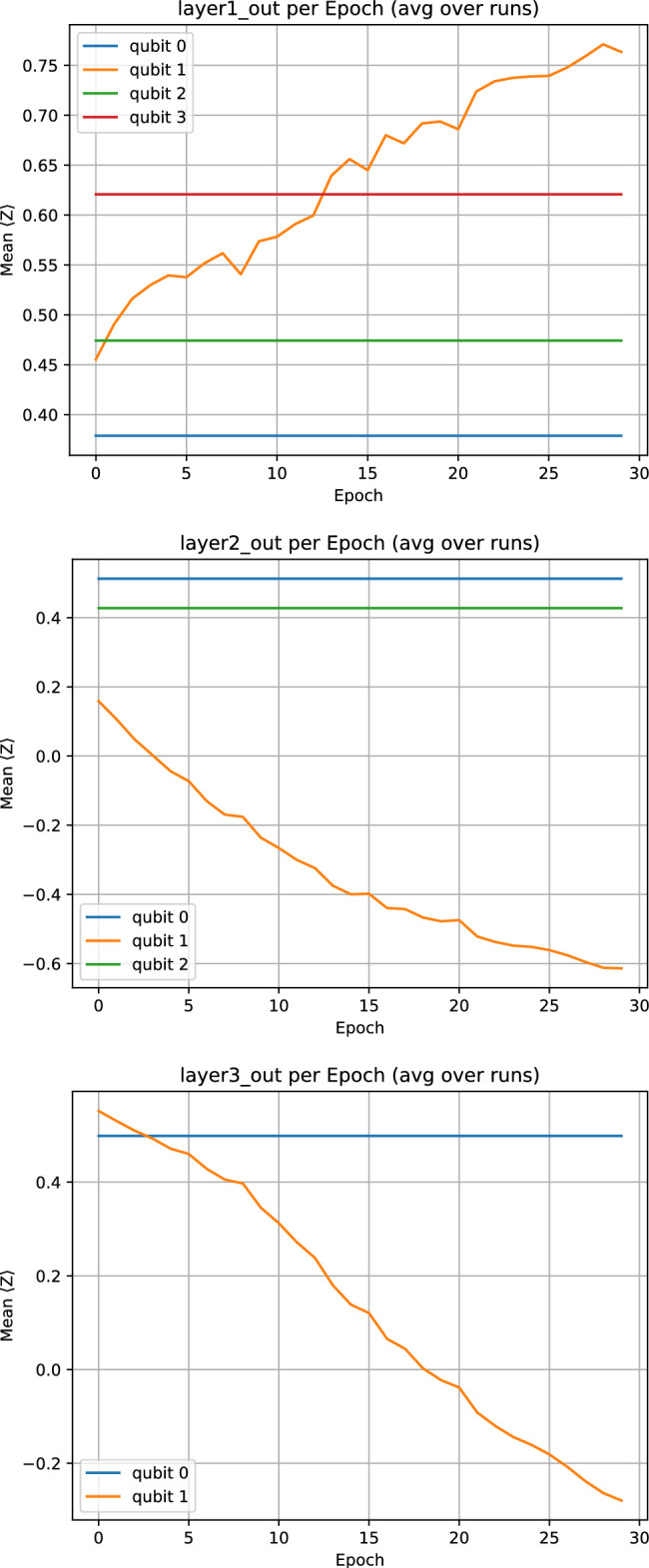
Mean $\langle Z\rangle$ per epoch for QT layers 1–3 (averaged across runs and over a fixed validation batch). Each curve corresponds to the channel of one QT block (labeled “qubit *i*” in the legend). Because only the blocks that lie on the decision path and ultimately influence the logit $\ell =z^{(3)}_2$ receive sustained gradient signal, their operating points move off saturation toward mid-slope regions and then stabilize; in the run shown these are the curves labeled “qubit 2” in each layer. The remaining blocks either do not feed into deeper layers or correspond to unused heads (e.g., $z^{(1)}_4$, $z^{(2)}_3$, $z^{(3)}_1$) and therefore experience almost no gradient and remain close to their initial operating points, which appears as nearly flat trajectories.

Figure 6 plots, for each epoch, the mean Pauli-*Z* expectation of the *channel* qubits in the QT blocks, averaged over a fixed validation batch; in each panel one curve corresponds to one QT block in the corresponding layer (labeled “qubit *i*” in the legend). Because of the wiring detailed in Sect. "Quantum transistor", only the QT blocks that lie on the effective decision path—those whose outputs are actually propagated forward and ultimately influence the logit $\ell =z^{(3)}_2$—receive sustained gradient signal from the loss. Blocks whose outputs are never used by deeper layers (e.g., $z^{(1)}_4$, $z^{(2)}_3$, $z^{(3)}_1$ in the current 4–3–2 layout) accumulate essentially zero gradient and therefore remain close to their initialization, which appears in the plots as nearly flat trajectories. Because the signal flow is index-preserving and the loss uses only $\ell =z^{(3)}_2$, only the QT with index $i=2$ in each layer lies on the computational path to the logit and therefore receives nonzero gradient. All other QTs are off-path (their outputs do not enter $\ell$) and thus remain close to initialization. The behavior in Fig. 6 is therefore determined by the wiring and the choice of logit, rather than by an emergent “routing” effect.

### Comparison to classical baselines

Table 3 summarizes mean test performance under the same subject-grouped folds, loss function, and calibration protocol, and using the same spectrogram windows as inputs. The CNN, Transformer, logistic, and tiny-MLP baselines operate directly on the full $40\times 12$ tensors (flattened to $D=480$ in the latter two cases), whereas the QT network acts only on the eight-dimensional contracted features derived from those tensors via Eq. (22). At the chosen configuration $p=5$, the QT stack therefore has 45 QT parameters plus $3848$ parameters in the classical contraction layer, for a total of $3893$ declared (executed-template) parameters. Under the present single-head, index-preserving wiring, only one QT per layer and one contraction coordinate influence the score used by the loss, so the effective trainable parameter count is $3p+481=496$ at $p=5$. The best CNN baseline uses approximately $1.6\times 10^{5}$ parameters under its HyperBand-selected configuration. A logistic-regression baseline with 481 parameters is comparable to the effective trainable size, and a tiny 1-hidden-layer MLP with $3857$ parameters is comparable to the executed-template budget; we report both comparisons for transparency. The logistic baseline attains test accuracy 0.8240 and test F1 0.8103, clearly below the QT network (accuracy 0.9600, F1 0.9310) despite having far fewer parameters. In contrast, the tiny MLP with essentially the same parameter budget as the QT reaches test accuracy 0.9680 and test F1 0.9683, i.e., it slightly outperforms both the QT network and the larger CNN/Transformer baselines in terms of F1. The Transformer_Encoder remains the most accurate model overall (accuracy 0.9794, F1 0.9642), but it does so with roughly two orders of magnitude more parameters than the QT. Given the QT’s strict resource profile (two qubits per block; constant two-CNOT entangling depth per block; nine data-dependent single-qubit rotations per block at $p=5$; Sect. "Quantum transistor"), these comparisons provide a fair, budget-matched view: the QT stack is competitive but does not yet surpass the strongest classical baseline at the same parameter scale. This directly answers *RQ3*.

**Table 3:** Summary of cross-validated performance (means over held-out folds; identical inputs, splits, losses, and calibration).

Model	Accuracy	F1
Quantum transistor network	0.9600	0.9310
Logistic regression (480$\rightarrow$1)	0.8240	0.8103
Tiny MLP (480$\rightarrow$8$\rightarrow$1)	0.9680	0.9683
CNN_Simple	0.9780	0.9615
Transformer_Encoder	**0.9794**	**0.9642**
CMMA (placeholder)	0.9721	0.9519

Significant values are in [bold]

**Fig. 7:**
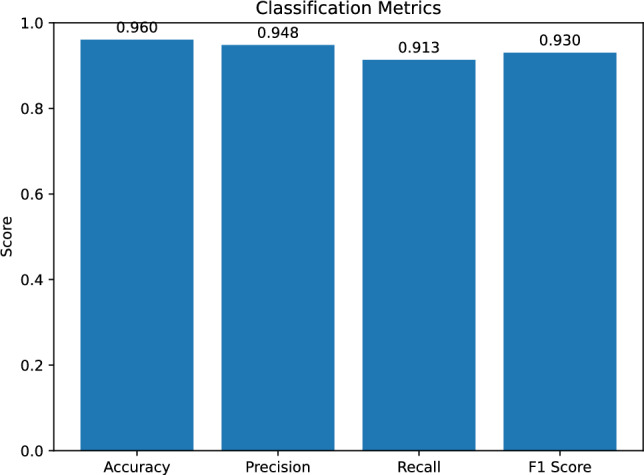
Aggregate classification metrics for the QT network (accuracy, precision, recall, F1) over held-out folds. Across-fold variability is negligible at the scale of this plot and is therefore not shown explicitly; fold-wise values and their standard deviations are reported in the text.

Figure 7 visualizes the aggregated accuracy, precision, recall, and F1 of the QT network over held-out folds under the subject-grouped evaluation protocol.

The class distribution is imbalanced (standing segments are more frequent), making decision thresholding consequential. Calibrating $\tau$ on each validation fold to maximize F1 yields the observed balance of errors: low false positives ($\approx 3.3$ per fold) and modest false negatives ($\approx 5.7$ per fold), which translates to precision $\approx 0.948$ and recall $\approx 0.913$ at test time. This trade-off is consistent with the QT’s transfer curve: under the multiplicative noise model of Eq. (23), the score distributions contract but preserve ordering, so the validation-chosen $\tau$ remains near-optimal on test folds. Qualitatively, the ROC curves in Fig. 5 show that the calibrated operating points lie on a region where small changes in $\tau$ do not dramatically alter F1, indicating a robust margin.

Each QT with $p=5$ comprises (in the generic two-wire template) two CNOTs for the biasing interaction (via the two-CNOT $\textrm{CRY}$ synthesis when $\textrm{CRY}$ is not native), nine data-dependent single-qubit rotations on the channel for the pre-bias stack, and a small number of fixed single-qubit gates for the bias synthesis (Sect. "Quantum transistor"). In the fixed-control instantiation studied here, a compiler may simplify the controlled bias to a single-qubit rotation, in which case these two-qubit gates are not required; we retain the two-qubit mapping as a transparent upper bound consistent with the standardized template. A forward pass through the full network uses at most eight concurrent qubits (Layer 1) and $2 \times (4 + 3 + 2) = 18$ entangling gates if layers are executed sequentially. Exact gradients via the parameter-shift rule require 2*p* shifted evaluations per QT (Eq. (21)); with nine QTs and $p = 5$, a single optimizer step entails 90 shifted circuits plus the unshifted forward, all at constant entangling depth. These counts match the released implementation and support reproducibility and hardware mapping.

From an application point of view, we do not report a direct numerical comparison against previously published gait-recognition studies because such comparisons are typically confounded by differences in sensor configuration (instrumented socks vs. inertial units, walkway systems, or depth cameras), population (multiple-sclerosis vs. healthy subjects or mixed cohorts), annotation protocols, and evaluation schemes (e.g., random vs. subject-grouped splits, window definitions, and decision thresholds). Instead, we adopt what we consider a more informative strategy and benchmark the QT stack against strong in-paper classical baselines that operate on exactly the same spectrogram windows, with the same grouped cross-validation and calibration pipeline. This allows us to interpret the reported F1 $=0.9310$ and accuracy 0.9600 as the performance of a compact, hardware-lean quantum layer relative to well-understood classical models under strictly controlled experimental conditions, rather than as headline numbers across incomparable datasets.

### Answers to the research questions (RQ1–RQ4)

For clarity and continuity, we restate here the research questions introduced earlier in the manuscript. The study was structured around four guiding questions. RQ1 concerns whether a standardized, two-qubit Quantum Transistor (QT) block can realize a meaningful transistor-like nonlinearity with stable, differentiable gain characteristics. RQ2 examines how such QT blocks should be organized-both in depth and in fan-in-to provide expressive yet resource-efficient stacked architectures. RQ3 evaluates whether a QT-based network, under a strictly subject-aware validation protocol, can achieve competitive generalization performance relative to strong classical baselines. RQ4 investigates which block-level design choices, including the bias configuration, the number of pre-bias rotations, and the pooling of multi-block outputs, most strongly influence the trade-off between expressivity, stability, and hardware cost.

With these questions restated for the reader, the following subsections present the empirical findings and discuss how each research question is addressed by the results.

RQ1 (Gating and amplification with stable gradients). *Answer: Yes.* QT behaves as a saturating nonlinearity with a controllable small-signal slope $g_q(0)=-\kappa _1\sin \phi$ (Eq. (27)). End-to-end training was stable across HyperBand trials (validation F1 $\approx 0.90$ by epoch 6), and the per-layer $\langle Z\rangle$ trajectories move off saturation towards mid-slope operating regions (Fig. 6). The exact parameter-shift gradients (Eq. (21)) maintained constant-depth evaluations and did not exhibit gradient collapse.

RQ2 (How to stack QTs under resource constraints). *Answer: 4–3–2 is Pareto-efficient in our gain budget.* Under identical block semantics ($p=5$, bias-last), the deep variant 8–4–2–1 was computationally impractical and provided no early-validation benefit, whereas the shallow variant 2–1 is ineffective. The intermediate layout 4–3–2 balances computation and generalization and is therefore adopted (see Sect. "Results and analysis" for ablation). This is consistent with the Jacobian-product and supports our guidance to prefer local re-uploads and bias-placement over additional depth or fan-in, while avoiding inter-QT entanglement.

RQ3 (Competitiveness vs. classical baselines with identical protocol). *Answer: Competitive but below the best classical baselines at matched parameter budgets.* With identical spectrogram windows, grouped splits, loss, and per-fold threshold calibration, the 4–3–2 QT stack trails both the strongest lightweight and the strongest high-capacity classical models; see Table 3. A tiny one-hidden-layer MLP with essentially the same parameter budget as the QT network ($3\,857$ vs. $3\,893$ parameters) reaches test F1 0.9683 and accuracy 0.9680, while a logistic-regression baseline with only 481 parameters attains test F1 0.8103 and accuracy 0.8240. The Transformer encoder, with roughly two orders of magnitude more parameters, achieves accuracy 0.9794 and F1 0.9642. Because the CNN, Transformer, and lightweight MLP baselines all see the full $40\times 12$ tensors whereas the QT network only sees their 8-D contractions, the performance gap remains a conservative upper bound on what would be observed if all models were restricted to the same low-dimensional interface (Sect. "Classical baselines and data collection process"). The depth extremes (8–4–2–1 and 2–1) did not close the gap (Sect. "Results and analysis"). The QT therefore offers a compact, analyzable footprint, albeit below state-of-the-art classical performance.

RQ4 (Impact of block-level design choices). *Answer: Clarified by ablation; key levers remain.* Among intra-block choices we *did* sweep, the number of pre-bias rotations (data re-uploads) mattered: HyperBand selected $p=5$ over $p\in \{2,3,4\}$ without increasing entangling depth per block. Depth and fan-in also had marked effects: a deeper 8–4–2–1 stack increased runtime by one to two orders of magnitude (epoch 1 $\sim \!3.78{\times }10^4$ s; subsequent epochs $\sim \!8.1{\times }10^3$–$1.40{\times }10^4$ s) and showed no early validation gains, while a shallow 2–1 stack reduced compute but underperformed the 4–3–2 layout (mean accuracy 0.954 vs. 0.960, mean F1 0.918 vs. 0.931). These results support our design guidance to prioritize local expressivity via re-uploads and bias-last semantics over additional depth or fan-in, and to avoid inter-QT entanglement. Two levers not ablated here remain promising: (i) making the bias angle $\phi$ trainable to place operating points where transconductance is largest ($g_q(0)=-\kappa _1\sin \phi$), and (ii) combining multiple QT heads of last - layers (or a small classical head) to improve statistical efficiency at constant qubit count.

## Discussion

The standardized Quantum Transistor (QT) block is designed so that the full pipeline-from feature extraction to calibrated decisions-can be implemented on real devices with a small, backend-portable circuit template. However, we emphasize the scope of the present study: all reported quantitative results are obtained via simulation (statevector / analytic evaluation), and we do not include a cloud-QPU (or in-lab) hardware execution in this manuscript. This choice is deliberate for two reasons. First, the specific instantiation evaluated here is the non-entangling special case that is efficiently classically simulable, so a hardware run would primarily quantify device-specific noise/shot overhead rather than probe an entanglement-enabled regime. Second, our main contribution is the *standardization* of a block-level primitive (ports, gain/saturation semantics, and a fixed compilation pattern) and its end-to-end integration with grouped validation and threshold calibration, which we can validate reproducibly on simulation while exposing all structural details to the reader. To make this concrete, the y Material provides a complete PennyLane reference implementation of the QT primitive and the full gait-processing pipeline (QNodes, parameter-shift gradients, grouped splits, and calibration), together with the manifest-level configuration used in the experiments. The QT port contract fixes a single real-valued input $s\in [-1,1]$ and a single scalar output $y(s)=\langle Z_t\rangle$; the forward map is unitary-plus-measurement and the backward map is parameter shift. Because each block obeys the same manifest (axes, order, bias-last semantics, and readout), software can assemble and unit-test stacks without per-circuit special cases, while a compiler/runtime can schedule identical instruction templates across devices. This contract also underwrites calibration: the midpoint $y(0)=\cos \phi$ and the small-signal slope $g_q(0)=-\kappa _1\sin \phi$ (Eq. (27)) can be verified by a two-point probe. In short, we make the standardization narrative executable via the released PennyLane prototype and an explicit gate-level mapping, while leaving full on-hardware evaluation (queueing, calibration drift, and finite-shot effects at scale) to future work.

From the viewpoint of many-body entanglement, our current QT stack occupies the extreme low-entanglement corner of the design space. Because each QT acts on a fresh pair of qubits and, in the present instantiation, the controlled bias does not generate entanglement, the overall *n*-qubit state factorizes across blocks and layers; all coupling between QTs happens through the classical scalars *y*(*s*) that are passed between layers. This is a deliberate engineering trade-off: it yields shallow, hardware-lean circuits with predictable gradients, but it also means that the architecture cannot exploit highly entangled *n*-qubit states that underlie many proposals for quantum advantage. We therefore do *not* claim that the present QT network is more expressive than generic, strongly entangling variational ansätze; instead, our results should be read as a lower bound on what can be achieved with a strictly local, almost single-qubit primitive. More entangling QT variants-for example, with data-dependent control qubits shared across channels or with explicit inter-QT entanglers-would strictly enlarge the function class representable by a fixed number of qubits and gates, at the cost of deeper circuits and potentially more severe optimization pathologies. Exploring this trade-off, and comparing the present architecture against such entangling alternatives on the same dataset, is an interesting direction for future work but lies beyond the scope of this study.

It is important to emphasize that the reported end-to-end performance is achieved by a *hybrid* model: a shallow classical contraction followed by a QT stack. We do not claim that the F1 score of the full system is attributable “purely” to the quantum layers. We also do not report an attribution baseline that replaces the QT stack with a simple classical classifier (for example, logistic regression) trained on the same contracted features *h*. In addition, because the present readout uses a single score, only one propagated chain through the executed stack carries nonzero gradient; we therefore temper any interpretation of these results as “stacked template” multi-path learning and treat pooling or multihead readouts as future work. The classical front-end contributes a learned linear projection and normalization of the spectrogram into eight channels, while the QT layers supply transistor-like gating and saturation via bounded trigonometric maps. A strict attribution of performance between these two components would require additional ablations (e.g., replacing the QT stack by a purely classical head operating on *h*, or freezing *W* while varying the QTs), which we have not performed here due to computational cost and the scope of the study. We therefore position our results as evidence that a compact QT stack, when paired with a minimal classical contraction, can participate meaningfully in an end-to-end learning pipeline on real data; quantifying the exact contribution of the QT layers relative to the classical front-end is an interesting direction for future work and we now state this limitation explicitly.

On real backends, stability comes from *bounded* depth and *predictable* compilation. Each QT uses one two-qubit biasing interaction (a native $\textrm{CRY}$ or its two-CNOT synthesis) after all data-dependent single-qubit rotations on the channel; this bias-last layout (Sect. "Quantum transistor") decouples data re-uploads from entangling costs. For $p=5$, one block incurs two CNOTs and nine data-dependent single-qubit rotations on *t*, independent of native one-qubit bases after compiler fusion. Because the measured observable is always *Z* on the channel, device noise contracts the transfer curve by a scalar factor $\lambda _Z$ (Eq. (23)) rather than distorting it, preserving the order of the scores and simplifying field calibration. These invariants-gate counts per block, output semantics, and noise contraction-allow backend teams to (i) budget CNOTs per shot and per layer, (ii) cache optimal placements for the repeated two-CNOT pattern, (iii) implement fast-path parametric sweeps for the few scalars that govern small-signal behavior, and (iv) ship conformance tests within tolerance on any device. In effect, the QT block provides a hardware-facing *interface specification*, not just a modeling idea.

Design lessons framed as a standardization roadmap:

1. *Expose bias as a first-class parameter.* The small-signal slope depends on $\phi$ via $g_q(0)=-\kappa _1\sin \phi$ (Eq. (27)); a standardized block should optionally treat $\phi$ as trainable and advertise its allowed range and resolution in the manifest so compilers can quantize angles without breaking gradients.
2. *Define a block manifest.* Each QT instance should declare axes $=(y,x,z)$ for the base triplet, the number of re-uploads $p-3$, the bias angle phi, the readout meas = $Z_t$, and the s-order (all data rotations first, bias last). This enables vendor-agnostic serialization and reproducible replays.
3. *Ship conformance tests.* Minimal device tests should confirm the midpoint $y(0)=\cos \phi$, the small-signal derivative from a symmetric two-point probe around $s=0$, and invariance of the transfer curve shape under repeated executions (up to a scalar $\lambda _Z$), instantiating Eq. (23).
4. *Resource contracts by construction.* Because a QT has constant entangling depth (two CNOTs in the non-native case) and a fixed readout, a stack can declare layer budgets as simple counts (e.g., $2\times (4+3+2)=18$ entanglers per forward pass), easing scheduling and enabling compile-time feasibility checks.
5. *Make threshold calibration part of the spec.* The pipeline should include per-fold validation calibration of the decision threshold $\tau$; since *y* is monotone-contracted by noise (Eq. (23)), keeping this step explicit yields stable test-time operating points across devices.
6. *Prefer local expressivity over extra entanglers.* When extending expressivity, add re-uploads on *t* before adding new two-qubit interactions; this preserves compilation regularity and aligns with the bias-last semantics already implemented.

The full stack-classical contraction (Eq. (22)), three QT layers with $(\eta ^\star ,p^\star )=(4.318\!\times \!10^{-4},5)$, parameter-shift gradients (Eq. (21)), and validation-threshold calibration-trained reliably: best trials crossed validation F1 $\approx 0.90$ by epoch 6 and improved smoothly thereafter. In fresh grouped 3-fold testing, the QT network achieved accuracy 0.960 and F1 0.931 with a consistent error profile (low $\textrm{FP}$, modest $\textrm{FN}$), while keeping the qubit footprint low (8 concurrent qubits at most) and the entangling budget explicit (18 per end-to-end forward when executed sequentially). These results, together with the gate/gradient accounting in Sect. "Quantum transistor", substantiate the claim that the QT abstraction is *practically* viable: it trains with exact gradients, calibrates cleanly, and compiles deterministically to short circuits.

As shown in Table 3, the parameter-matched tiny MLP and the higher-capacity Transformer outperform the present QT stack on this classical gait dataset under identical splits and calibration. We therefore do *not* recommend the current QT instantiation as a drop-in replacement for state-of-the-art classical models when the sole objective is maximal F1 on this benchmark. The intended value proposition is instead an engineering one: QT blocks provide a repeatable, low-depth, backend-portable quantum-layer interface whose behaviour (midpoint, gain, saturation, and monotone noise contraction) is analytically characterizable and verifiable via simple conformance tests. This enables hardware/software co-design and deployment reasoning in terms of predictable qubit/two-qubit-gate/shot budgets, stable calibration procedures, and iteration via block-local changes (e.g., making $\phi$ trainable) without refactoring the surrounding pipeline. These properties can matter in scenarios where a quantum co-processor is present for other reasons—most notably when upstream data are already quantum (quantum sensing / quantum readout pipelines) or when co-locating inference near a cryogenic or embedded quantum front-end reduces classical data movement and enforces stable latency envelopes. Importantly, we do not claim latency/energy advantages here and we do not measure them in this manuscript; we identify these as the appropriate axes for future evaluation of standardized QT primitives on real hardware, alongside accuracy.

## Conclusion and future work

This work demonstrates the *viability* of a standardized, hardware-conscious hybrid classical–quantum learning pipeline centered on the Quantum Transistor (QT) block. We specified a two-qubit, biasable, differentiable primitive with fixed ports and bias-last semantics; derived its transfer curve and small-signal gain; provided exact gradients by parameter shift (Eq. (21)); and embedded the block in a three-layer network with a simple classical contraction front-end (Eq. (22)). The resulting system trains stably under a clear gain budget, calibrates its decision threshold on validation folds, and compiles to short, repeatable circuits with constant entangling depth per block (and, in the present non-entangling instantiation, is efficiently classically simulable). We therefore do not claim quantum advantage; the contribution is the standardized block interface and its transparent hybrid integration. In grouped 3-fold tests on gait dynamics, the QT stack reached accuracy 0.960 and F1 0.931, trailing both a tiny MLP baseline with a comparable parameter budget (F1 0.9683) and a larger Transformer baseline (F1 0.9642); accordingly, we do not advocate the present QT instantiation as a performance-optimal replacement for classical gait classifiers. Instead, we view these results as an end-to-end validation that a standardized QT block can be trained, calibrated, and compiled deterministically under a transparent resource/shot budget, which is the prerequisite for future studies that evaluate QT-ready implementations on hardware and along hardware-relevant metrics (latency, energy, and sensor-level integration) in addition to accuracy.

The central contribution is the *standardization* itself. By fixing ports, gate order, readout, and differentiation rules, a QT library can support (i) vendor-agnostic serialization and compilation; (ii) conformance tests that verify midpoints, slopes, and noise-contraction behavior; (iii) resource declarations that make scheduling and feasibility checks trivial; and (iv) calibration procedures that remain valid under moderate device drift thanks to Eq. (23). This elevates quantum model building from ad-hoc circuits to components with predictable *system* behavior.

From a hardware-design vantage point, and while this manuscript does not present an execution on a cloud QPU, a standardized QT can be treated as a fixed *macro-instruction* with a known two-qubit signature (native $\textrm{CRY}(\phi )$ or a two-CNOT synthesis) and a small set of scalar knobs ($\{\kappa _j\}$ and $\phi$). This admits several concrete advantages: (i) *layout and scheduling*-tiling nearest-neighbor (*g*, *t*) pairs and caching a single two-CNOT template reduces compilation variance and enables compile-time CNOT budgets; (ii) *calibration economy*-device conformance can be driven by two-point probes of the midpoint *y*(0) and small-signal slope $g_q(0)$, while slow drift is summarized by a single contraction factor $\lambda _Z$ (Sect. "Quantum transistor"), lowering the dimensionality of routine recalibrations; (iii) *pulse-level fusion*-pre-bias single-qubit stacks on *t* can be merged into short frame changes, leaving only the fixed bias interaction, which shortens depth and improves duty cycle; (iv) *native-gate opportunities*-couplers can be engineered toward a calibrated $\textrm{CRY}_\phi$ family with stable amplitude control, turning the QT bias into a first-class hardware primitive; and (v) *parameter broadcast*-because many QTs share the same instruction template, microarchitectures can amortize angle updates (the $\kappa _j s$ waveforms) across blocks. Together, these aspects point to “QT-ready” devices with predictable performance envelopes and faster field calibration.

Immediate, compatible extensions are straightforward. First, make the bias $\phi$ learnable per block to place operating points where transconductance is largest; the manifest should expose its range and quantization. Second, pool all last-layer QT outputs with a small classical head or an additional QT reducer, which increases statistical efficiency without changing the qubit budget. Third, enrich encodings at constant entangling cost by adding re-uploads on *t* and light classical mixing between layers, leveraging the Fourier–harmonic view. Fourth, explore one additional QT layer while keeping the two-CNOT-per-block contract. Fifth, characterize robustness on hardware via the conformance suite (midpoint, slope, and monotone contraction) and apply lightweight error mitigation where it preserves the QT semantics. Finally, formalize a vendor-agnostic QT registry that ships manifests, compilation metadata, and reference tests, so that devices can advertise “QT-ready” status with quantitative tolerances.

In closing, the QT block converts quantum-model design into an engineering discipline: small, composable, biasable units with closed-form transfer curves, exact gradients, and fixed compilation patterns. This is the level at which hardware and algorithm teams can co-design in earnest. As these standardized primitives mature-and as modest architectural enhancements close the remaining accuracy gap-quantum learning pipelines can move from artisanal prototypes to reproducible, deployable *systems*.

Finally, the classical–quantum mapping in Table 1 turns device-level concepts (biasing, transconductance) into first-class *software-visible* knobs; together with a fixed two-qubit signature, this is precisely the structure needed for QT-ready hardware and compiler pipelines.

## Supplementary Information

Supplementary Information.

## Data Availability

The wearable-sensor gait data underlying this study were collected from human participants and therefore constitute potentially identifiable biometric information. In accordance with the approvals of the Ethics Committee of Getafe University Hospital (CEIm) and the Technical Committee of the Universidad Politécnica de Madrid, the raw time-series data are not publicly available. The code required to reproduce the QT circuits, the training/evaluation pipeline (grouped splits and per-fold threshold calibration), is publicly available at https://zenodo.org/records/18559151. Derived, de-identified features and evaluation metadata may be made available from the corresponding author upon reasonable request and subject to institutional approval and an appropriate data-use agreement.
