# Supplementary material for: Standardized quantum transistor block enables differentiable learning on gait dynamics
Source: Sci Rep. 2026 Feb 18;16:9506. doi: 10.1038/s41598-026-40424-7 (PMC13004870; doi:10.1038/s41598-026-40424-7)
Supplement: Supplementary file 1 — Supplementary Information. [file 41598_2026_40424_MOESM1_ESM.pdf]

Supplementary Material: Standardized Quantum Transistor Block Enables Differentiable Learning on Gait Dynamics

Javier Villalba-Díez[1,2*] and Joaquín Ordieres-Meré[3]

[1]Fakultät Wirtschaft, Hochschule Heilbronn, Max-Planck-Str.39, Heilbronn, 74081, Baden-Württemberg, Germany.
[2]Department of Mechanical Engineering, Universidad de La Rioja, Logroño, 26004, Spain.
[3]Escuela Técnica Superior de Ingenieros Industriales, Universidad Politécnica de Madrid, Madrid, 28006, Spain.

*Corresponding author(s). E-mail(s): javier.villalba-diez@hs-heilbronn.de;

1 Introduction

This supplementary document provides detailed experimental configurations and comparative ablation studies for the Quantum Transistor (QT) and the budget-matched classical MLP baseline discussed in the main text. The results presented here are derived from the attached replication notebooks: `quantum_transistor_ablation_Q.ipynb` and `quantum_transistor_ablation_Classic.ipynb`.

2 Model Architectures and Hyperparameters

2.1 Quantum Transistor (QT) Stack

The QT stack is instantiated as a three-stage network designed for differentiable learning on normalized scalar features[cite: 50, 616].

- **Layer 1:** 4 QT blocks (8 qubits total)[cite: 52, 617].
- **Layer 2:** 3 QT blocks (6 qubits total)[cite: 53, 618].
- **Layer 3:** 2 QT blocks (4 qubits total)[cite: 54, 619].

The optimal configuration identified through HyperBand optimization included **3 parameters per block** and a learning rate of 1.56×10^{-4}.

2.2 Classical MLP Baseline

A Tiny MLP was designed as a baseline matched to the parameters to contextualize QT performance. The architecture utilizes a standard feed-forward structure optimized via HyperBand:

- **Layer configuration:** [16, 48, 64] units.
- **Hyperparameters:** Learning rate: 0.0032, Weight Decay: 1.69×10^{-5}, Dropout: 0.5.

3 Performance Comparison on Unseen Data

The models were evaluated on a held-out test split and an unseen supervised test dataset to assess generalization.

Table 1 Model Comparison on Held-out Test Split

Model	Accuracy	F1-Score	Precision	Recall
Quantum Transistor (QT)	0.960	0.931	–	–
Classical Tiny MLP	1.000	1.000	1.000	1.000

4 Ablation Results: The Switching Effect

The notebooks demonstrate a unique ”snap” behavior in the QT layer, where the classification accuracy suddenly jumps from random superposition (50%) to near-perfect performance once the circuit finds an optimal interference pattern.

4.1 Confusion Matrices

Below are the confusion matrices for the final test phase:

- **Quantum Model:** Achieved near-perfect separation on calibrated thresholds, with mean F1 of 0.931.
- **Classical Model:** On the held-out test split, the MLP achieved a perfect confusion matrix with zero misclassifications: $\begin{bmatrix} 64 & 0 \\ 0 & 61 \end{bmatrix}$.
